# Spatial Stochastic Dynamics Enable Robust Cell Polarization

**DOI:** 10.1371/journal.pcbi.1003139

**Published:** 2013-07-25

**Authors:** Michael J. Lawson, Brian Drawert, Mustafa Khammash, Linda Petzold, Tau-Mu Yi

**Affiliations:** 1 Department of BioMolecular Science and Engineering, University of California, Santa Barbara, California, United States of America; 2 Department of Computer Science, University of California, Santa Barbara, California, United States of America; 3 Department of Mechanical Engineering, University of California, Santa Barbara, California, United States of America; 4 Department of Biosystems Science and Engineering, ETH-Zürich, Basel, Switzerland; 5 Department of Molecular, Cellular, and Developmental Biology, University of California, Santa Barbara, California, United States of America; University of Notre Dame, United States of America

## Abstract

Although cell polarity is an essential feature of living cells, it is far from being well-understood. Using a combination of computational modeling and biological experiments we closely examine an important prototype of cell polarity: the pheromone-induced formation of the yeast polarisome. Focusing on the role of noise and spatial heterogeneity, we develop and investigate two mechanistic spatial models of polarisome formation, one deterministic and the other stochastic, and compare the contrasting predictions of these two models against experimental phenotypes of wild-type and mutant cells. We find that the stochastic model can more robustly reproduce two fundamental characteristics observed in wild-type cells: a highly polarized phenotype via a mechanism that we refer to as spatial stochastic amplification, and the ability of the polarisome to track a moving pheromone input. Moreover, we find that only the stochastic model can simultaneously reproduce these characteristics of the wild-type phenotype and the multi-polarisome phenotype of a deletion mutant of the scaffolding protein Spa2. Significantly, our analysis also demonstrates that higher levels of stochastic noise results in increased robustness of polarization to parameter variation. Furthermore, our work suggests a novel role for a polarisome protein in the stabilization of actin cables. These findings elucidate the intricate role of spatial stochastic effects in cell polarity, giving support to a cellular model where noise and spatial heterogeneity combine to achieve robust biological function.

## Author Summary

Cell polarity is the fundamental process of breaking symmetry to create asymmetric cellular structures. It is an open question how randomness (stochasticity) in the cell hinders or helps cell polarity. In this work, we focus on the ability of yeast cells to sense a spatial gradient of mating pheromone and respond by forming a projection in the direction of the mating partner. A key element is the polarisome, which is at the tip of the mating projection. We introduce the first model of polarisome formation in yeast. The model is well-supported by experimental data. We perform modeling to explore the role of noise in the formation of the polarisome. By running simulations with and without noise, we arrive at the surprising conclusion, that gradient-dependent polarization is enhanced by stochasticity. Both the tight localization (amplification) and the ability to respond to directional change of the input (tracking) are enhanced by stochastic dynamics, resulting in a more robust behavior. Mutants in which key polarisome proteins have been deleted exhibit broader, noisier polarisome than the wild type. The mutant phenotype is accurately captured by our stochastic simulations. These results demonstrate the importance of stochasticity in the study of cell polarity.

## Introduction

Cell polarity is a classic example of symmetry-breaking in biology. In response to an internal or external cue, the cell asymmetrically localizes components that were previously uniformly distributed. This polarization underlies behaviors such as the chemotaxis of motile cells up chemoattractant gradients and asymmetric cell division during development [1], [2].

In *Saccharomyces cerevisiae*, a haploid cell (**a** or 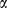) senses a spatial gradient of mating pheromone from its partner and responds by producing a mating projection toward the source. The peptide pheromone binds to a G-protein coupled receptor which activates the heterotrimeric G-protein. Free 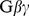 recruits Cdc24 to the membrane where it activates Cdc42. The spatial gradient of activated Cdc42 (Cdc42a) is used to position the polarisome, which generates the mating projection [3]–[5]. The role of actin-mediated vesicle transport in the establishment and maintenance of Cdc42a polarity is an area of active research [6], [7], however we only focus on the downstream components.

The polarisome, located at the front of the cell, helps to organize structural, transport, and signaling proteins [8], and guides polarized transport and secretion along actin cables. The polarisome's function is conserved in eukaryotes, and analogous scaffold complexes are responsible for such diverse structures as focal adhesions and synapses [9].

**Figure 1:**
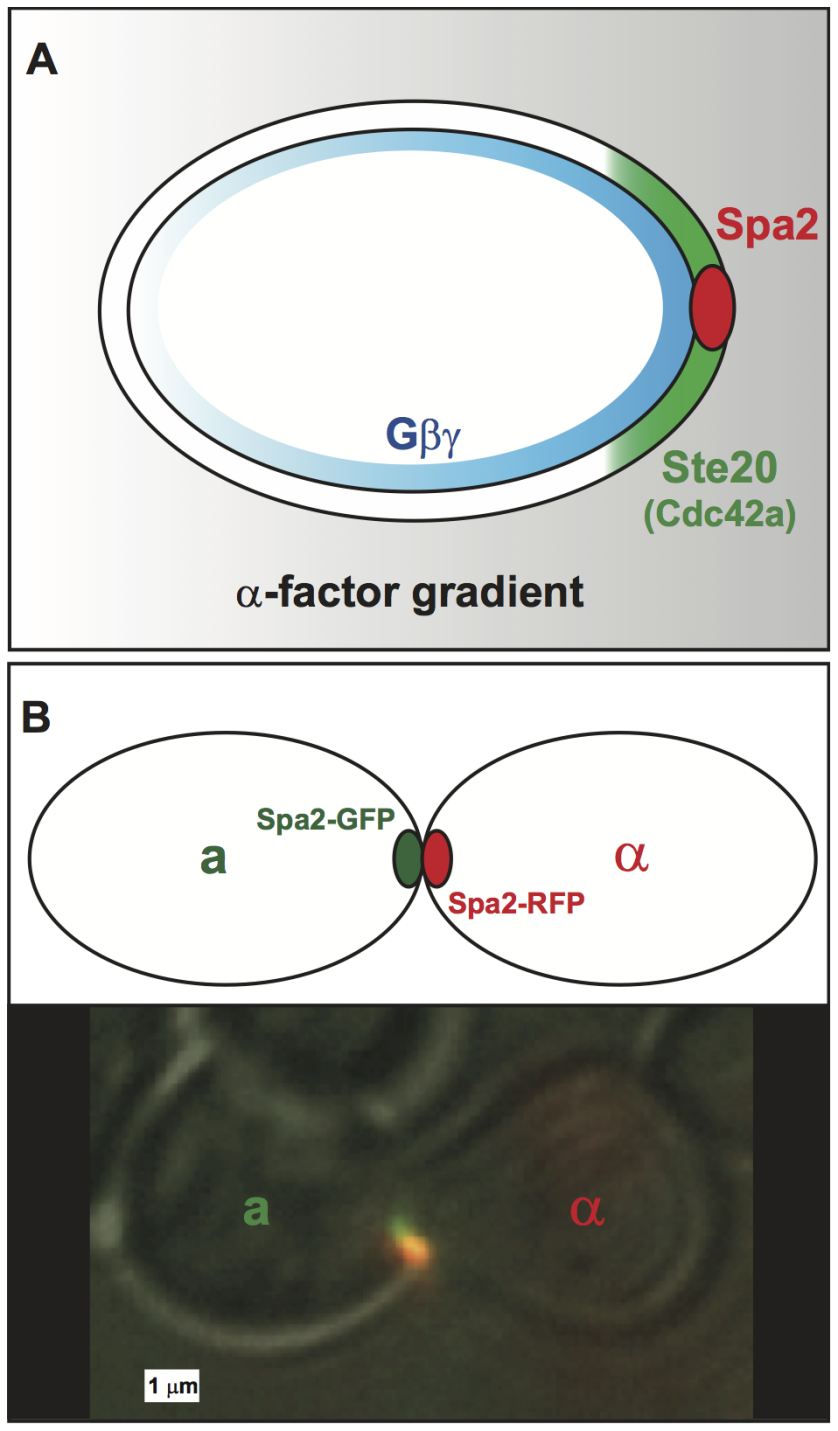
Spatial amplification in cell polarity during yeast mating. (**A**) Spatial amplification occurs in stages during cell polarization in yeast. The external spatial gradient of 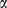-factor is shallow (gray), and it generates a comparable gradient of free 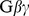 on the cell membrane. This initial internal gradient induces a polarized cap of active Cdc42 (green) which in turn localizes the tightly condensed polarisome (red) to the front of the cell. In this manner, a shallow external gradient is amplified to a steep internal gradient. (**B**) A schematic and microscopy image of two mating yeast cells with aligned punctate polarisomes. The polarisomes are labeled with Spa2-GFP (**a**-cell) and Spa2-RFP (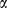-cell). During mating the polarisomes at the tip of the mating projection are tightly localized and seek out one another until they are aligned and adjacent. When the projections meet the membranes and polarisomes fuse, and mating occurs.

A striking feature of the polarisome is its narrow localization at the tip of the mating projection. The process of transforming a shallow external gradient into a steep internal gradient (i.e. all-or-none) of protein components is a process we term spatial amplification, and is a significant challenge to understand and model in cellular polarization [10], [11]. In yeast, this polarization occurs in steps through successive stages of the mating pathway from the extracellular gradient of 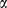-factor (gray in Fig. 1A) to the more pronounced polarization of 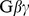 (blue) to the crescent cap of active Cdc42 (green) to the punctate polarisome at the front of the cell (red) [12].

The two main components of the polarisome are Spa2 and Bni1. Spa2 is an abundant scaffold protein important for structural cohesion of the polarisome; Bni1 is a formin that initiates the polymerization of actin cables, which direct vesicles to the front of the cell [5]. In the absence of Bni1, the mating projection forms slowly and is misshapen [13]. In the absence of Spa2, the mating projection adopts a broad appearance and the polarisome is no longer a single punctate entity [14]–[16]. In both loss-of-function mutants, mating efficiency is drastically reduced. One hypothesis is that proper mating requires the alignment of punctate polarisomes (Fig. 1B). Indeed, mutants that exhibit abnormal polarisome dynamics often also exhibit decreased mating performance [13], [14], [17]. These data are consistent with the view that a tightly localized polarisome is critical for efficient mating.

A second key feature of the mating projection and polarisome is the ability to track a gradient that may be changing direction. Mathematical modeling of cell polarization highlights the potential tradeoff between amplification, which produces the tight polarization, and tracking of a moving signal source [18], [19]. Positive feedback is one way to achieve amplification, but this feedback can impede the ability to follow a shift in signal direction. Recent studies have shown that fine-tuned modulation of positive feedback can lead to proper polarization and chemotaxis [11], [20], whereas disruption of the positive feedback results in defective polarization [21]. Most of these studies have relied on deterministic models of spatial dynamics. An important question is how stochastic spatial dynamics affect cell polarity, and more specifically how noise affects the amplification/tracking tradeoff.

The impact of noise and stochastic dynamics on signal transduction, protein interaction networks, and gene regulation has gained broad recognition [22]. Examples range from the stochastic lysis-lysogeny decision in phage lambda [23] to transcriptional noise in yeast [24] to morphogen gradient noise in *Drosophila* [25] to stochastic dynamics in the human brain [26]. As a result, many stochastic models of biological systems have been developed [27] including one of the yeast mating pheromone pathway [28]. In fact, continuous deterministic models (governed by ordinary differential equations) represent a limiting case of more accurate discrete stochastic models (governed by the chemical master equation) [29].

However, most of these models have been non-spatial, in which the system is considered well-mixed. Recent results have shown the need for spatial stochastic models. For example, Fange and Elf [30] have modeled spatiotemporal oscillations of the MinD and MinE proteins during cell division in *E. coli*. They found that stochastic simulations were essential to reproduce the phenotypes of certain mutants such as avoiding bistability when the cell is spherical, and forming nucleated clusters of MinD in PE^−^ mutants. Similarly, Altschuler et al. [31] have modeled cell polarity in yeast using stochastic spatial dynamics. In this system, polarization was induced by overexpressing constituitively-active Cdc42. Their stochastic models of cell polarization involving self-recruitment [32] and actin nucleation with directed transport [33] have highlighted the important role of spatial stochastics in initiating and maintaining spontaneous polarization. In these studies, the authors focused on polarization in the absence of a cue, and did not investigate the amplification or tracking of a gradient.

We present a mathematical model of Cdc42a-gradient induced polarisome formation. To our knowledge this is the first such model. The model is well-supported by experimental data, and we discuss the process of obtaining the parameters from experimental data. There are only two free parameters in the model, and we explore this space via extensive parameter sweeps. Comparing the results of stochastic and deterministic models with equivalent structure, stochastic simulations reveal better and more robust tradeoffs between spatial amplification and signal tracking. In particular, spatial stochastic effects contribute to tighter polarization, an effect we refer to as *spatial stochastic amplification*. In addition, only the stochastic model can reproduce both of these characteristics of the wild-type (WT) phenotype, as well as the multi-polarisome phenotype of the 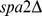 mutant. Finally, our work suggests a novel role for a polarisome protein in the stabilization of actin cables.

## Results

### Model Description

We have constructed a mathematical model of the formation of the yeast polarisome. Focusing on the final stage of the mating system, our model takes the broad Cdc42a distribution on the membrane as the input and seeks to produce a narrow polarisome as the output. We sought a simple model that captures the essential dynamics while limiting the size of the parameter space for model analysis. The chemical reactions that make up our model structure were simulated both stochastically and deterministically. The following two subsections describe the model structure and the parameter estimation based on our biological data. For further model details see Sections 2.3 and 3 in Text S1.

### Model Structure

**Figure 2:**
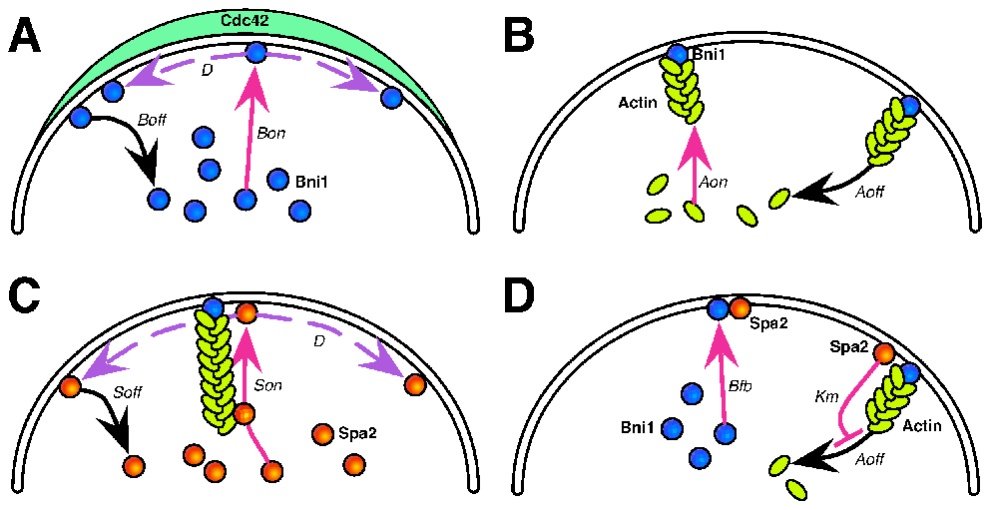
Diagram describing yeast polarisome model. (**A**) Input driven recruitment of cytoplasmic Bni1 by membrane bound active Cdc42 (Cdc42a). (**B**) Bni1 on the membranes nucleates and polymerizes actin cables. (**C**) Actin cables direct transport of Spa2 from the cytoplasm to the membrane. (**D**) Spa2 provides positive feedback as it recruits cytoplasmic Bni1 to the membrane and inhibits actin depolymerization.

Fig. 2 describes our model of the formation of the punctate yeast polarisome in response to a cue: the broader polarization of activated Cdc42 (Cdc42a). The polarisome is a complex structure consisting of at least five different proteins [5]. The two primary functions of the polarisome proteins are structural (scaffolding) and catalytic (nucleation of actin cables). Spa2 is the most abundant scaffold protein, thus to simplify our model we aggregated the scaffold species into Spa2. Bni1 is the formin responsible for actin cable formation during the mating response [13], and so we aggregated the actin nucleation dynamics into Bni1.

The input to the model is the experimentally measured membrane profile of Cdc42a, and the output is the spatial profile of Spa2. Note that in the simulations, Cdc42a is not a dynamic state variable. However, the direction of the Cdc42a polarization can be shifted as a change in input. To estimate the input profile, we averaged the fluorescence intensity of a Cdc42a reporter, Ste20-GFP, over multiple cells (Fig. S14 in Text S1). In the model, Cdc42a recruits Bni1 to the membrane. Using a combination of two-hybrid and molecular pull-down data, Evangelista et al. demonstrated that active Cdc42 directly binds Bni1 [13]. Bni1 on the membrane nucleates actin cable assembly, producing a positive feedback loop via Spa2, which is delivered to the membrane by transport along the actin cables. For simplicity, we do not explicitly model the actin polymerization process, but instead model actin cables attaching to and detaching from the membrane (Fig. 2), thus combining actin initiation and polymerization into a single event.

There are two positive feedback loops in the model. First Spa2 recruits Bni1 to the membrane. There is experimental evidence for Spa2 binding Bni1 [34]. Second, a polarisome component inhibits actin depolymerization so that more Spa2 can be transported to the membrane along actin cables. This is accomplished via an inhibition term possessing a Michaelis-Menten type form (see Table S3 in Text S1), which is valid in a stochastic setting [35]. We constructed two versions of the model structure because the exact mechanism of actin stabilization is not known: one in which Bni1 inhibits actin depolymerization (B-model), and one in which Spa2 inhibits actin depolymerization (S-model). In the main text we will focus on the S-model; analogous results can be found for the B-model in the SI. The strength of the first positive feedback loop can be adjusted via the parameter 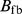, and the strength of the second positive feedback loop by the inhibition constant 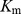. The second positive feedback loop is a model hypothesis motivated by prior modeling results [33]. However, we note that Yu et al. [36] have shown that during yeast budding, polarization is accompanied by more stable actin cable dynamics. In addition, formins in other organisms can facilitate actin bundling at higher concentrations [37]. Finally, Spa2 interacts with a number of accessory proteins including Myo2 which exhibits synthetic lethality with Tpm1, which binds and stabilizes actin cables [38]. Table S3 in Text S1 lists the equations that we use to describe the model structure.

For each of the two possible model structures, we employed two modes of simulation. The first uses discrete stochastic kinetics for diffusion and biochemical reactions. The second uses a continuous deterministic formulation, i.e. the familiar partial differential equations for reaction-diffusion systems (for more details on both formulations see Materials and Methods).

### Parameter Estimation

We used our simple model structure to estimate most of the reaction rates and diffusion constants from our *in vivo* data. This section provides a summary of the full calculations described in Section 3 in Text S1 for the S-model; similar calculations were made for the B-model.

**Figure 3:**
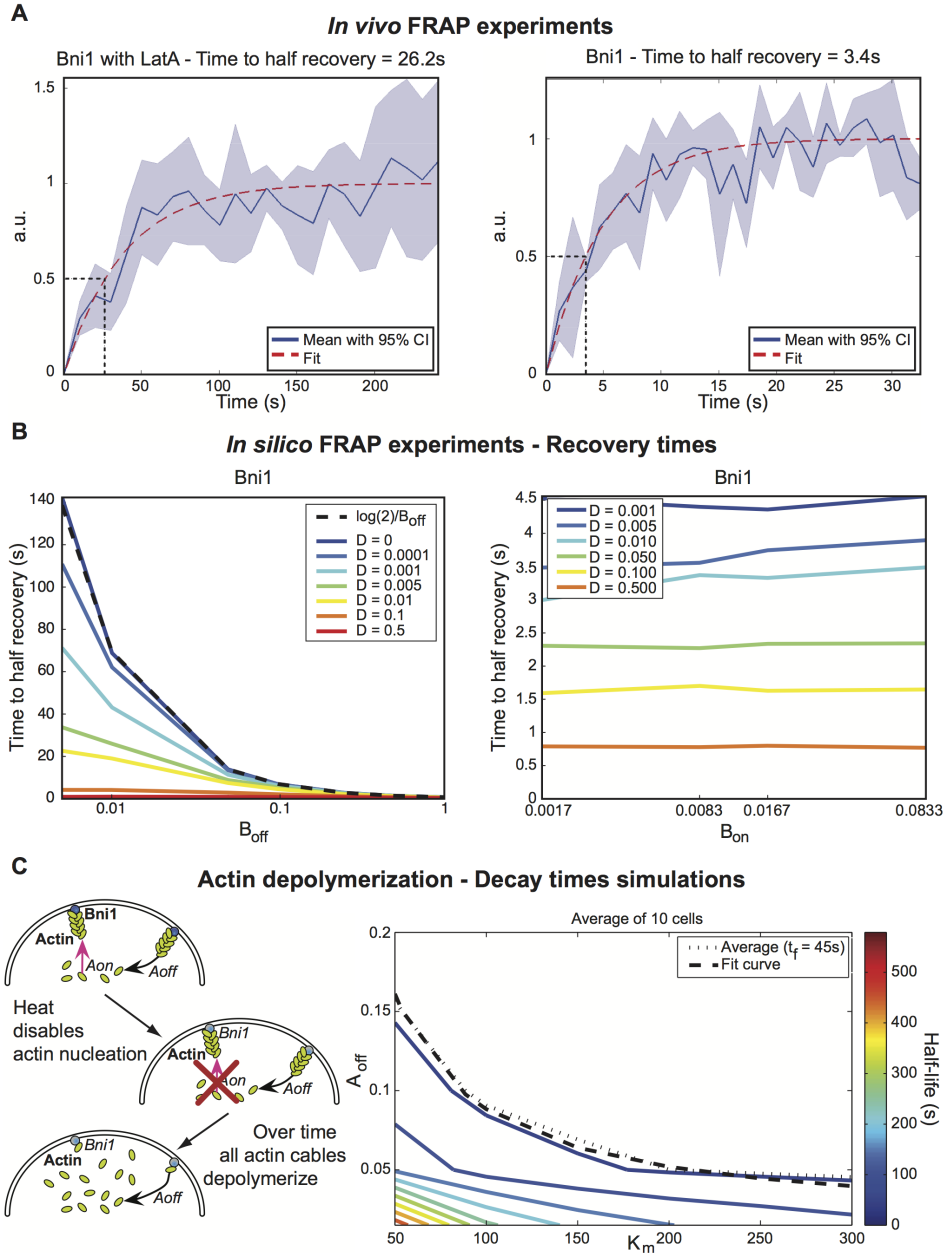
Parameter estimation from experimental data including FRAP. (**A**) Experimental FRAP recovery curves for Bni1 (Solid blue line: average of 5 experiments. Light blue area: 95% confidence interval around average. Dashed red: fit to exponential). The fit curve is used to determine the time to half-recovery, therefore the data is normalized by the maximum value of the fit curve. Notice that the WT curve has a much shorter time to half recovery than the LatA-treated curve (time to half recovery indicated by dashed black lines). Also see video S1. (**B**) FRAP simulation time to half recovery for varying diffusion rates and 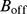 (left) and 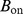 (right). There is no change with 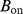, while for 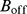 as 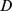 goes to zero the curves approach the theoretical no-diffusion limit (dashed black). **Also see video S2.** (**C**) Left: Cartoon illustrating the Bni1 temperature sensitive mutant experiment performed in [41] and simulated in this paper. Right: Phase plane of actin cable half-life (color-coded) as a function of 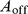 and 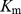 (simulation of the experiment in [41]), with the curve representing 45 s (dotted black) and our model fit (dashed black). This phase plane represents the average of those generated for initial conditions corresponding to 10 different observed cells.

Similar to the approach of Marco et al. [33], we performed fluorescence recovery after photobleaching (FRAP) experiments (Fig. 3A) in the presence and absence of the actin depolymerization agent Latrunculin A (LatA). With LatA treatment we observed a greatly extended recovery time for Bni1, implying that actin-dependent recycling was increasing the recovery rate (compare Fig. 3A FRAP recovery curves (curves represent average of 5 experiments with 95% confidence interval)). From the LatA FRAP recovery curves, we directly estimated the membrane diffusion coefficient for Bni1 to obtain 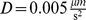. Because we did not exclude actin-independent mechanisms of Bni1 membrane removal, the measured diffusion coefficient represents an upper bound, however this value is consistent with the slowest of those measured for membrane proteins in [39]. The membrane localization of Spa2 depends on actin, thus we could not perform the identical analysis on Spa2. Instead, we set the diffusion coefficient for Spa2 to be the same as Bni1, a protein similar in size.

We derived an analytic expression relating the recovery half-time to the off-rate using a simple version of our model in the limit of no diffusion [40]: 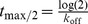. Then we calculated 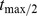 from FRAP simulations of the full model using a range of values for 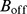 and the diffusion constant 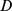 (left panel of Fig. 3B). As 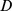 goes to zero, the simulations converge toward the no-diffusion limit analytic expression (Fig. 3B). These results do not depend on a specific value for 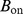 (right panel of Fig. 3B). From the curves in Figs. 3A and B, and the measured value of 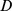, we were able to estimate 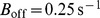. The same procedure was performed for Spa2, with the result 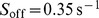 (see Fig. S2 in Text S1). These values correspond to a region of parameter space in which the polarisome reaction kinetics are faster than the diffusion kinetics for typical yeast membrane proteins.

From the steady-state levels of Spa2 (approximately 90% on the membrane, see Fig. S4 in Text S1) and 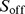, we estimated 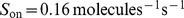. The fact that approximately 20% of Bni1 is on the membrane cannot uniquely determine the 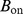 parameter, as Bni1 is delivered to the membrane in two ways: recruitment by Cdc42a (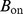) and binding by Spa2 (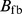). Instead, our analysis produced a linearly constrained relationship between 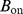 and 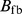 (Equation S8 in Text S1).

Finally, we estimated the rate constants for actin polymerization/depolymerization. We made use of data from Evangelista et al. [41], in which the authors induced Bni1 loss-of-function using a temperature-sensitive allele, and then measured the time course of the number of cells possessing actin cables. Assuming that actin depolymerization is an exponential decay process, the half-life of a single cable is the same as the half-life of a population of cables. Therefore, we can use the decay curve in [41] to estimate a depolymerization rate for individual cables. We fit the data with an exponential decay curve and determined that the time to half actin depolymerization was approximately 45s. *In vitro* experiments by Carlier et al. [42] agree with this timescale for actin depolymerization. In our model, depolymerization depends on both a basal rate 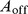 and an inhibition term containing the constant 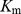, representing Spa2 inhibition of actin depolymerization. After substituting the actin cable half-life, we obtained the following equation for the total actin depolymerization rate: 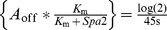. We note that a similar expression holds for the B-model. Using the above we estimated the polymerization rate constant 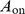 to be 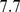
e-5 molecules^−1^s^−1^.

We performed simulations of the actin depolymerization experiment [41] to derive a simpler expression for the relationship between 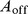 and 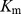. Starting with probability distributions of Spa2 and Bni1 taken from fluorescence data as initial conditions, we varied 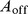 and 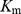 and calculated the actin cable half-life. This was repeated on data from 10 cells. The right panel of Fig. 3C shows a phase plane of decay times as a function of the two parameters. From this graph, we were able to obtain a direct relationship between 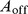 and 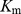 (Fig. 3C, dashed black).

In summary, we used experimental data to identify 6 parameters in the model, reducing the free parameter space to two dimensions: the 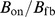 ratio and the (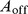, 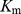) relationship. A third undetermined parameter is the total number of actin cables. In the deterministic simulations variation of this parameter did not affect polarization, thus it was explored separately. We have selected values for the two free parameters based on the models ability to reproduce the spatio-temporal characteristics of our *in vivo* data (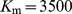, 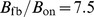). We use these values as our nominal parameter set.

### Spatial Stochastic Amplification

**Figure 4:**
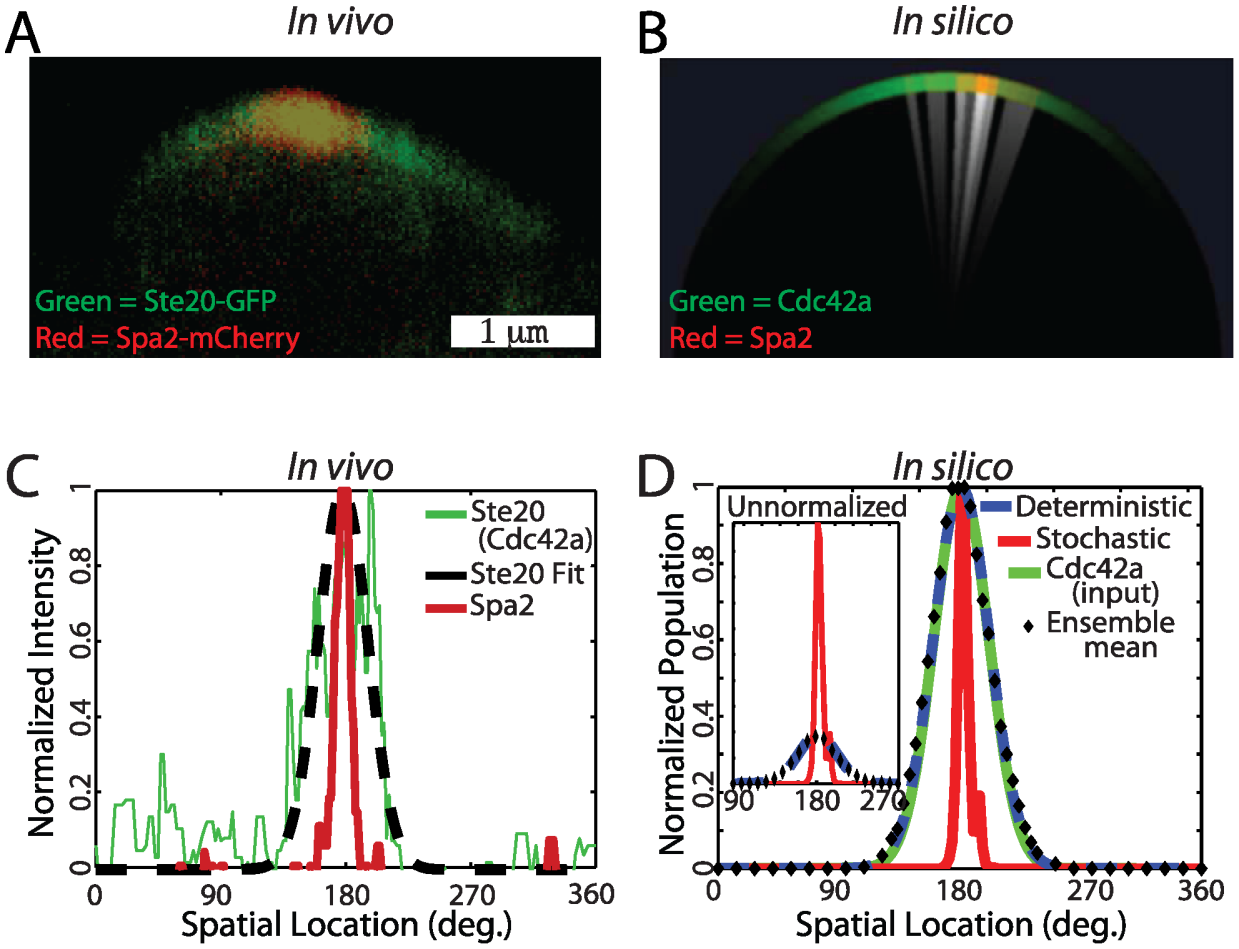
Punctate Polarization (Green: Cdc42a, Red: Spa2). (**A**) Yeast cells treated with 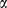-factor show the wider Cdc42a (marked by Ste20-GFP) and tighter Spa2 polarization. (**B**) Visualization of a stochastic realization of the polarisome model (white indicates regions with actin cables attached to the membrane). (**C**) Normalized fluorescence intensity membrane profiles of Ste20-GFP and Spa2-mCherry from a yeast cell undergoing polarisome formation (Green: Ste20 (Cdc42a). Dashed black: Ste20 fit. Red: Spa2). Ensemble mean experimental data is in Fig. S19. (**D**) Normalized membrane intensity profile from stochastic and deterministic realizations of polarisome model, the Cdc42a input, and the mean output of a stochastic ensemble (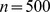). Inset: Absolute membrane intensity profile from stochastic and deterministic realizations of polarisome model, and Spa2 ensemble mean. (Red: Spa2 Stochastic. Dashed blue: Spa2 deterministic. Black diamond: Spa2 ensemble mean. Green: Cdc42a (input)).

A striking feature of the yeast polarisome is its tight localization compared to the broader polarization of Cdc42a (Fig. 4A). To characterize this polarization experimentally, we used Ste20-GFP as a fluorescent reporter for Cdc42a (an alternative reporter Gic2-208-GFP produced similar results, see Fig. S8 in Text S1). In pheromone-induced cells, it spanned a full width at half maximum (FWHM, see Materials and Methods) of approximately 48° on the membrane (averaged over multiple cells, see Fig. 4C, S14 in Text S1). The punctate polarisome was marked by Spa2-mCherry, which localized to a region of FWHM approximately 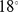. Cdc42a directs the localization of the polarisome by binding polarisome components such as Bni1 [5]. As described above, we refer to spatial amplification as the transformation from a broader input polarization (Cdc42a) to a narrower output polarization (Spa2).

The parameter estimation described in the previous section left our model with two remaining degrees of freedom: 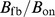 and (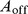, 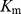). Exploring this space we found that, for any given parameter set, the stochastic model always produced tighter polarization than the deterministic model. We refer to this cue directed noise-driven emergent behavior as *spatial stochastic amplification*. This is illustrated in Fig. 4D for our nominal parameter set, and this behavior is observed across parameter space (see Fig. S15 in Text S1). The sharp stochastic peaks sample a range of directions within the Cdc42a profile similar to what is observed experimentally (Fig. S9 in Text S1, videos S7 & S8), whereas the deterministic peak is stationary. As a result, for the stochastic model the average of the measured widths is narrow (see Fig. S14B), while the width of the average over an ensemble of time points is broad (black diamond curve in Fig. 4D). We found that the deterministic simulation of the model overlaid the ensemble average of stochastic trajectories (see Fig. 4D). Moreover, as we demonstrate below, increasing the stochasticity of the dynamics results in increased amplification.

### Tracking of a Dynamic Input

**Figure 5:**
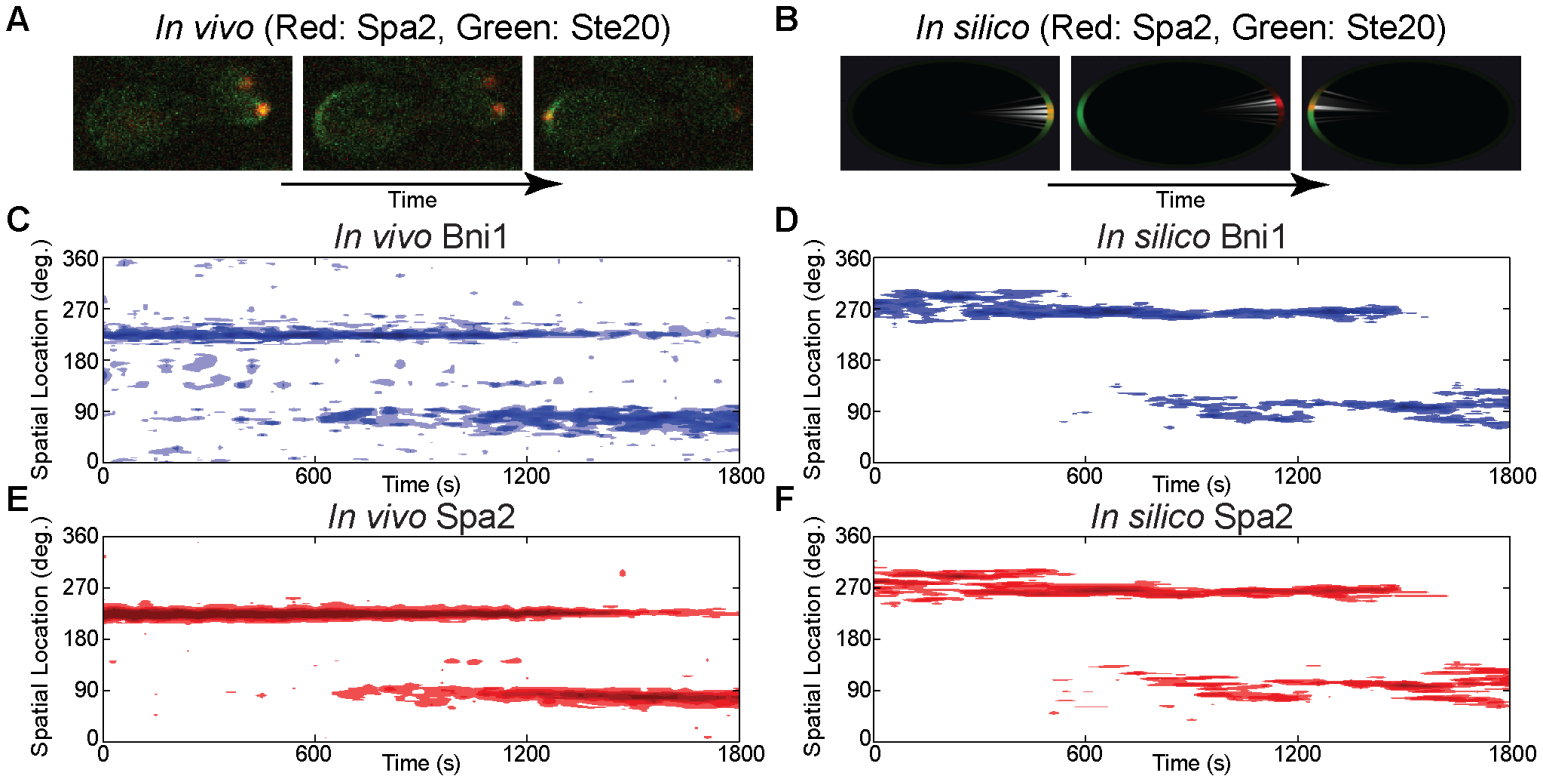
Polarisome tracking of directional change in Cdc42a (Green: Ste20 (Cdc42a), Red: Spa2, Blue: Bni1). **Left:** *In vivo* data. **Right:** *In silico* data. **Top row:** In both the cell (**A**) and the simulation (**B**), the Cdc42a profile shifts first, followed by the the polarisome (indicated by Spa2). **Middle and bottom rows:** Spatial dynamics of Bni1 (**C, D**) and Spa2 (**E, F**) during polarisome tracking of Cdc42a. Note that the time scale of polarisome switching is similar between *in vivo* and *in silico* experiments, especially in the 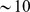 minute overlap time when two polarisomes are present. **Also see videos S3 and S4.**

A second key performance objective that must be balanced against tight polarization is that the yeast polarisome must track a change in the direction of the input cue (Cdc42a). In yeast cells, a change in the 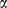-factor gradient direction results in a corresponding change in Cdc42a polarization. Similarly, extended exposure to isotropic pheromone will also induce a change in Cdc42a localization because of the oscillatory dynamics underlying the formation of multiple projections [16], [43], [44]. We imaged dual-labeled Ste20-GFP/Spa2-mCherry cells in 100 nM 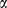-factor. Under both directional gradient (Fig. S10 in Text S1) and isotropic 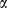-factor conditions (Fig. 5A), we observed that Cdc42a shifts its position to a new polarization site. After a delay (middle panel of Fig. 5A) this change was followed by the relocalization of the polarisome (right panel of Fig. 5A). We refer to the relocalization of the polarisome following a shift in Cdc42a orientation as successful tracking.

We also imaged cells with Bni1-GFP/Spa2-mCherry to observe the spatio-temporal dynamics of these two polarisome constituents during tracking. A typical time trace for Bni1 and Spa2 is shown in Figs. 5C and 5E respectively. We note that there is 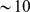 minute transition period during which the nascent second polarisome has begun to form while the initial polarisome still persists. The median characteristic time of this overlap from *in vivo* measurements of five cells was 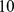 minutes (mean = 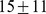, see Fig. S13 in Text S1).

In both Cdc42a/Spa2 (Fig. 5B) and Bni1/Spa2 (Figs. 5D and 5F) dynamics we found that our *in silico* experiments matched the characteristic spatio-temporal features of our *in vivo* experimental results. Fig. 5B shows that there is a delay in polarisome relocation after a switch in Cdc42a orientation. This is an indication of the need to be in a parameter regime that balances tight polarization (determined in part by 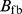) and the ability to successfully track the input signal (determined by 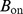), a tradeoff that will be discussed further in the following section.

Figs. 5D and 5F show that Bni1 and Spa2 populations in our *in silico* experiments reproduced the spatio-temporal polarisome characteristics noted in our *in vivo* experiments: 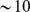 minute transition period in which two polarisomes are present. Analysis of an ensemble of 500 trajectories gave an median overlap time of 9.5 (mean = 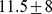) minutes (Fig. S13 in Text S1). This confirms that the polarisome dynamics produced by our model qualitatively and quantitatively agrees with what we observed experimentally.

Although the deterministic simulations were also able to exhibit the appropriate tracking behavior (Fig. S12 in Text S1), it was in a much less robust fashion. As we demonstrate below, the stochastic simulations can track for narrower polarisome widths and do not require fine-tuning of the parameter values.

**Figure 6:**
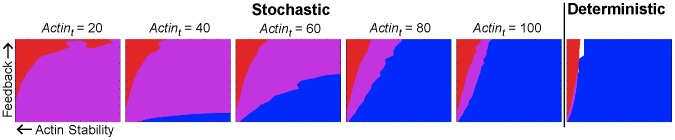
Six polarization phenotype space plots of 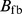/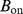 ratio versus 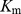. The first five panels show results from the stochastic model with varying 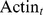 values of 20, 40, 60, 80, 100 (left to right); the final panel shows results from the deterministic model. 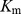 values (*x*-axis) range from 0 to 4000, 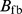/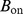 ratio (*y*-axis) ranges from 0 to 10. Blue indicates accurate tracking (>70% probability), red indicates narrow width (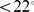 FWHM), purple indicates that both criteria are met and white indicates that neither criterion is met. As the number of actin cables is increased, the stochastic phenotype plots converge to the deterministic plot. Lower actin cable number confers a larger region where both criteria are satisfied, indicating that increased stochasticity leads to more robustness to parameter variation. For each plot, the 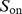 parameter was adjusted to maintain a constant flux of Spa2 to the membrane.

In Figs. 4 and 5 we compared a single stochastic realization with the deterministic simulation to allow comparison of individual trajectories. In the following section (Fig. 6) we show the average behavior of an ensemble of stochastic realizations and compare it to the deterministic simulation.

### Robustness to Parameter Perturbation

Previously, it has been hypothesized that there is a tradeoff between the amplified polarization and the ability to follow changes in signal direction [11], [45], [46]. We explored this hypothesis in the context of the polarisome system and investigated the effects of stochastic dynamics on the tradeoff. To accomplish this, we generated phase planes in parameter space for the deterministic and stochastic formulations of the S-model with 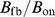 on the *y*-axis and 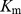 on the *x*-axis. We measured polarisome width and the ability to track a directional change. These plots demonstrate this tradeoff for both the stochastic and deterministic simulations (Fig. 6). Stronger positive feedback (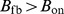) and a more stable polarisome (small 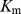) produced tighter localization. Because tracking requires the ability to sense a new input direction and shift the polarisome to the new site, greater input influence (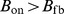) and less polarisome stability (large 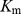) yielded better tracking.

To elucidate the effect of stochasticity on the polarisome system, we varied the total number of actin cables in the cell. Fig. 6 shows phase planes for various levels of total actin cables in the cell, from 20 to 100. We also adjusted 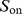 (rate of actin-mediated Spa2 delivery) to maintain the same flux of Spa2 to the membrane for different numbers of actin cables. In our model approximately half of the total population of actin is on the membrane, and thus the lower range of 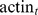 (total number of actin cables) values is consistent with the measurements made by Yu et al. [47]. For the deterministic model, varying the number of actin cables had no effect on polarisome dynamics (so long as the population was greater than zero) because partial differential equations treat protein populations as continuous, and thus the same profile was produced for all total actin populations. The dynamics of the stochastic model, on the other hand, was strongly dependent on actin cable number.

For the lower range of actin cables there were two striking differences between the performance of the stochastic and deterministic simulations: the stochastic model exhibited a larger overlapping region in parameter space in which both the amplification and tracking criteria were met (see Fig. 6, compare the purple region of the first and last panels). Additionally, the deterministic model displays an abrupt transition to tracking failure, whereas the stochastic model has a smoother tradeoff between polarisome width and the probability of successful tracking (Fig. S15 in Text S1). To illustrate this tradeoff, we examined the minimum possible width given successful tracking. For deterministic models the minimum width was 11.3° For stochastic models (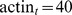), the minimum width depended on the strictness of the tracking criteria: at 75% tracking probability the minimum was 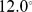, at 70% it was 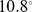, and at 50% it was 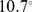. This provides further evidence that the stochastic dynamics of this system play a non-trivial role in polarisome formation.

As the number of actin cables was increased, we observed that the gap between stochastic and deterministic performance decreased. As we increase the number of actin cables, the stochastic phase plane increasingly resembles the deterministic one (see Fig. 6). The effect is striking both in terms of the individual regions of sufficient amplification (red) and tracking (blue) as well as in the region of overlap (purple). The convergence to deterministic behavior with larger populations is not surprising, given that deterministic models represent the large population limit of stochastic models. Large total actin cable populations reduce the intrinsic noise in the system, making the stochastic model behave deterministically. Thus, these data suggest that the number of actin cables determines the level of stochasticity in the polarisome system and that increased noise in the system confers robustness to parameter perturbations. Finally, we find all the above observations hold in both the S- and B-models (compare Fig. 6 and Fig. S16 in Text S1) in which either Spa2 or Bni1 inhibits actin depolymerization. Indeed, the simulations showed that the presence of polarisome protein mediated inhibition (i.e. 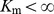) produced significantly superior amplification (for a given level of tracking) than the absence of the inhibition, suggesting a novel role for a polarisome protein in the stabilization of actin cables.”

### Stochastic Simulations Reproduce the Mutant Phenotype

**Figure 7:**
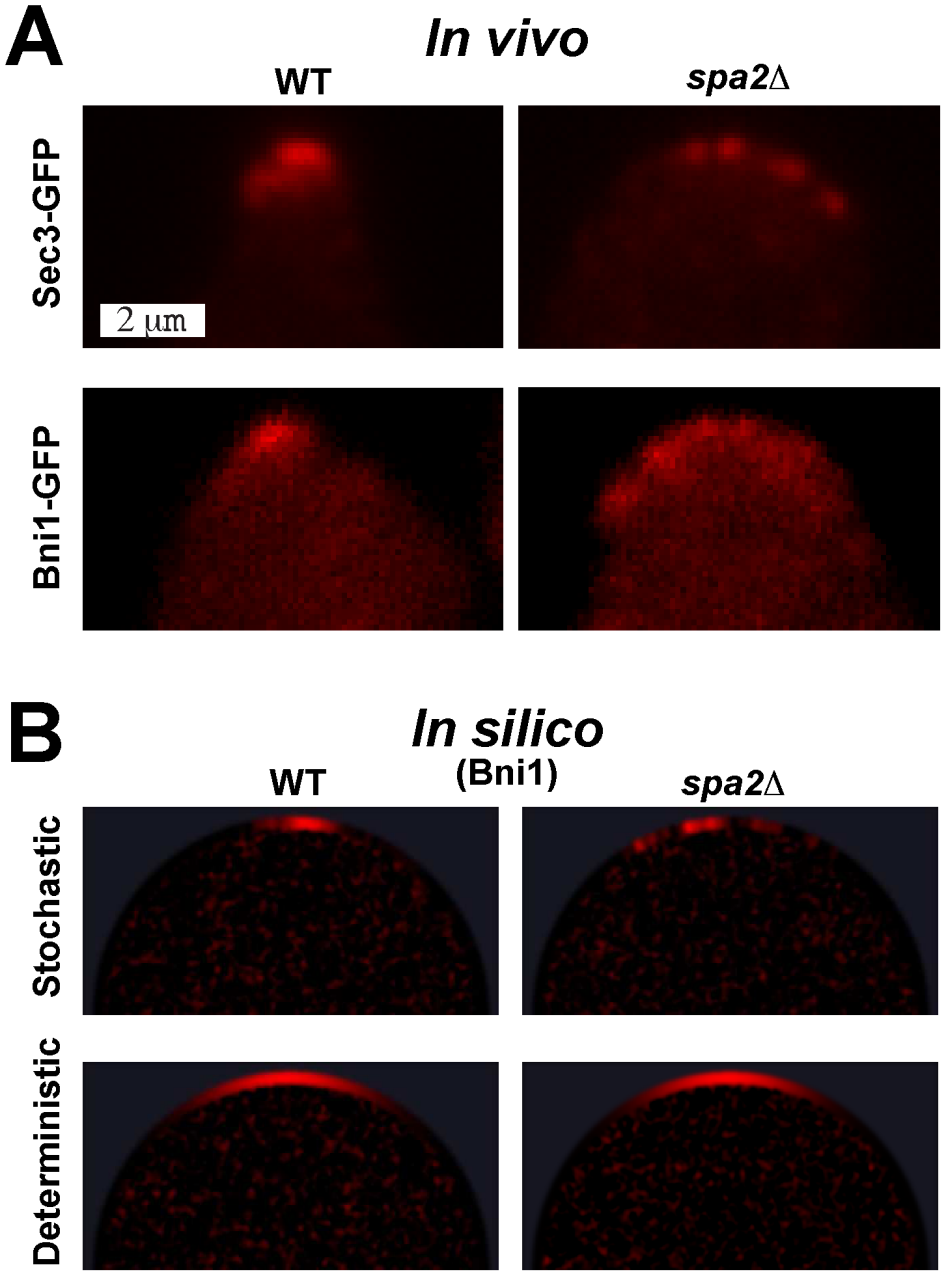
The multi-polarisome phenotype in 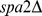 cells. **Columns:** WT phenotype (**left**), 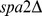 phenotype (**right**). (**A**) *In vivo* microscopy images of polarizing yeast cells marked with Sec3-GFP (top row) and Bni1-GFP (bottom row). Note the difference between the single punctuate polarisome (left) and the multi-polarisome phenotype (right). Also see video S5. (**B**) *In silico* snapshots of yeast polarisome simulations for both stochastic (top row) and deterministic (bottom row) models showing Bni1. Note that only the stochastic *in silico* model is able to match the *in vivo* multi-polarisome phenotype. **Also see video S6.**

We characterized the *in vivo* dynamic behavior of pheromone-induced 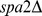 cells [14] compared to WT cells. In both, the polarisome was marked by the protein Bni1 tagged with GFP (bottom row of Fig. 7A). Because the low total population of Bni1 made visualization difficult, we also included mutant and WT images of cells containing the more abundant polarisome marker Sec3 tagged with GFP (top row of Fig. 7A). After a two hour treatment with 100 nM 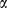-factor, WT cells possessed a polarisome that exhibited only moderate variability in spatial and temporal behavior (left column of Fig. 7). On the other hand, 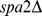 cells displayed a distinct phenotype in which multiple polarisome foci appeared (multi-polarisome phenotype), possessing a more dramatic noisy behavior and broader polarization (right column of Fig. 7A, videos S5 & S6). These results provide support for the Spa2-dependent positive feedback in the model.

We performed spatial stochastic and deterministic simulations for both WT and 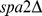 cells (Fig. 7B). In the stochastic simulations we were able to observe the dynamic behavior of the WT polarisome. More strikingly, the stochastic simulations were able to capture important aspects of the 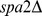 multi-polarisome phenotype in terms of multiple foci (top right panel of Fig. 7B), noisy behavior, and broader polarization. In contrast, deterministic simulations of the model could not reproduce the WT or 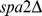 polarisome behaviors (bottom right panel of Fig. 7B). Specifically in the 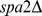 case, the deterministic simulations showed neither the presence of multi-polarisomes nor the noisy dynamic behavior. Of course it is important to appreciate that the absence of noisy dynamic behavior is not necessarily a defect of our model; it is a premise of the deterministic modeling formalism.

## Discussion

In this work we have constructed a simple model of the yeast polarisome, a classic example of cell polarity, focusing on the dynamics of the proteins Bni1 (a formin) and Spa2 (a scaffold protein). The parameters in the model were fit to experimental data including FRAP experiments performed on living cells (videos S1 & S2). We note that this is, the first mathematical model of the polarisome, and as such provides a valuable foundation for future studies of this system. In addition, our model suggests a novel role for a polarisome protein (i.e. Spa2 or Bni1) in the stabilization of actin cables, which we plan to test in the future.

Our *in silico* experiments have shown that stochastic dynamics produced qualitatively different results from deterministic dynamics. First, we found that *spatial stochastic amplification* provided tighter polarization across a range of parameters. Second, the intrinsic noise enabled better tracking given tight amplification, provided increased robustness to parameter perturbations, and better reproduced the qualitative searching behavior of the polarisome (see below). Finally, only the stochastic model was able to reproduce the 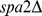 multi-polarisome phenotype.

This work builds upon and extends the previous work of Marco et al. [33] and Altschuler et al. [32] The key difference is that we focus on the polarisome and the physiological process of sensing and responding to an input gradient of Cdc42a, versus spontaneous polarization in the absence of a cue. However, in all cases, the research demonstrates the power of spatial stochastic dynamics to initiate, amplify, and adjust the polarity.

Similar to the work of Fange and Elf [30], we demonstrate that stochastic but not deterministic simulations can reproduce the phenotype of a mutant in which random spatial clusters appear. In yeast, the wild-type polarisome is a punctate structure that senses an input signal, but forms multiple foci when the Spa2-mediated positive feedback is diminished. In *E. coli*, the MinD protein is normally dispersed and undergoes oscillations, but forms random clusters when the positive feedback is increased (rate of spontaneous association with the membrane is decreased). In both cases, spatial clusters arise from the amplification provided by stochastic spatial dynamics.

**Figure 8:**
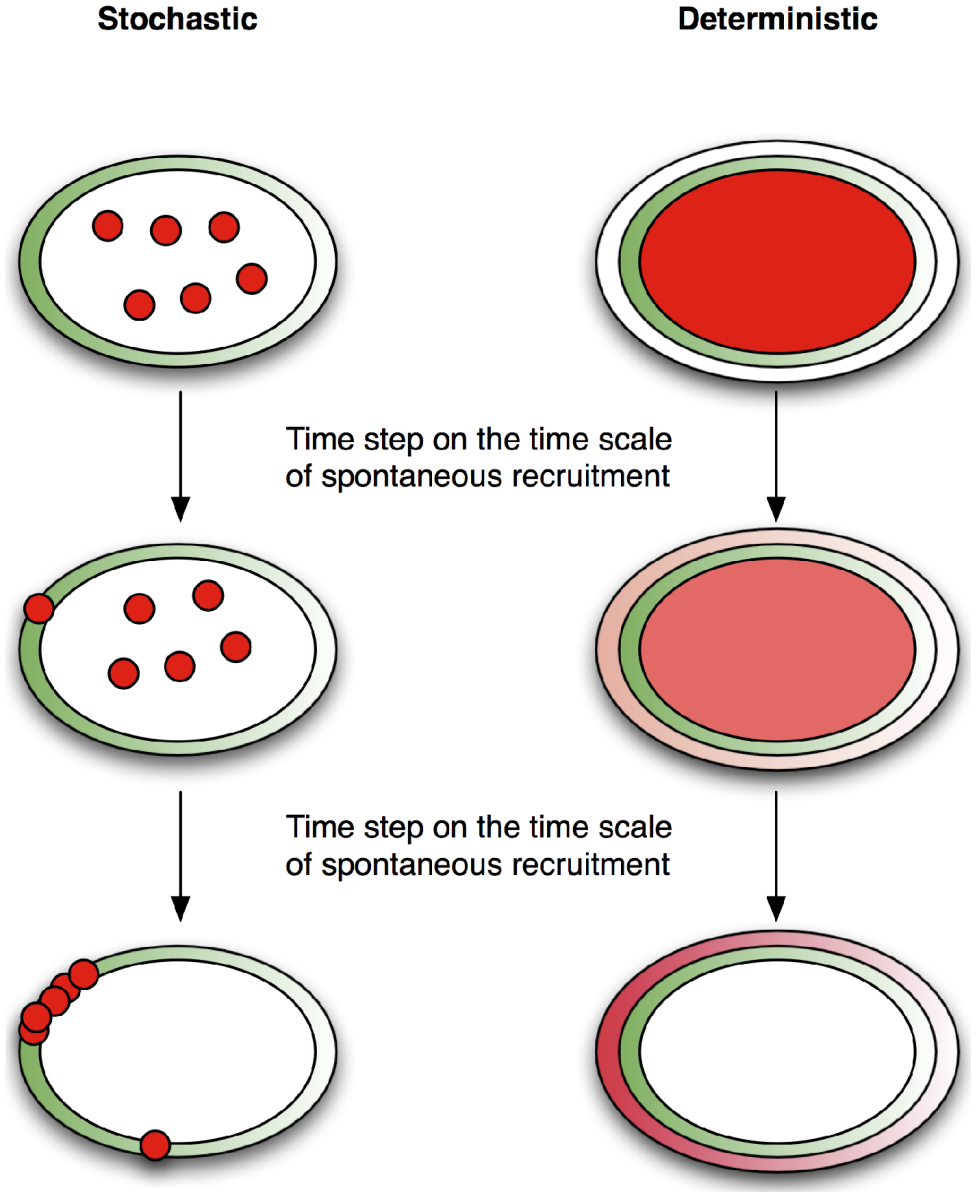
Stochastic versus deterministic polarization schematic time course. Green is the input Cdc42a profile, Red is the output (Spa2 or Bni1). **Top:** Initially, in both the stochastic and deterministic simulations, all of the output protein is in the cytoplasm. **Middle:** After a short time period one molecule has been recruited to the membrane. In the stochastic simulation this addition takes place in one discrete location, whereas in the deterministic simulation the addition is in a continuous concentration gradient along the membrane. **Bottom:** This difference in allocation of molecules results in differing final profiles. In the stochastic case, feedback has recruited most of the output protein to the location of the first addition, whereas in the deterministic simulation output protein has been added smoothly along the membrane, resulting in a smooth final distribution.

The two keys to understanding *spatial stochastic amplification* are the discreteness of molecules and the noisy nature of chemical systems. The discreteness of molecules dictates an integer number of proteins in a given location, so that the addition of a molecules is a unit step in population. If molecules were continuous in the sense of concentration, then there would be a smooth addition of molecules to the membrane. Fig. 8 explains the results of these essential differences. In both the stochastic and deterministic cases we begin at the top of the diagram with all of the Spa2 (or Bni1) in the cytoplasm. After some period of time the first Spa2 molecule in the stochastic simulation has moved to the membrane. By contrast, in the deterministic simulation, a concentration of Spa2 has been added to the membrane in a smooth distribution, in which the total membrane population is equivalent to one molecule of Spa2. Finally, the presence of Spa2 creates positive feedback. In the stochastic simulation this is only in one discrete location, however in the deterministic simulation, Spa2 exists in a spatially varying continuum across the membrane. In this way it becomes clear how stochastic simulation of the same parameter set, resulting in roughly the same membrane fraction of protein, produces tighter polarization than deterministic simulation. In the parameter regime where this difference is most notable the output and input shapes are decoupled. That is, the output polarization is the same regardless of input shape (e.g. gradient steepness), but its location is biased by the input profile. As is noted above, this process is related to the positive feedback induced symmetry breaking without a directional cue observed in [32].

The polarisome engages in a dynamic search process during mating projection formation and tracking. There are two aspects to this behavior. First, in a single projection, the polarisome scans within the polarized region of Cdc42a as the projection attempts to align with the gradient (Fig. S9 in Text S1). Second, during the formation of the second projection, the polarisome explores the new region before it becomes more tightly localized (see video S3). Both of these behaviors can be observed in the stochastic simulations (see videos S8 and S4).

As has been noted in [11], [45], [46] and is clear from the phase planes in Fig. 6, there is a tradeoff between tight polarization and tracking. Given tighter localization it might seem intuitive that the stochastic simulations would be less likely to track. Tight polarization generally requires stability and strong feedback. Reliable tracking requires instability and high input sensing. The noise in the system lends additional intrinsic instability to the system independent of feedback/sensing tradeoffs, allowing for reliable tracking for relatively tight polarization.

An important feature of robust biological models is that they do not require careful selection of parameters. When modeling bacterial chemotaxis, Barkai and Leibler [48], [49] demonstrated that the experimentally observed perfect adaptation was a structural property of their model, while alternative models required fine-tuning of the parameters to achieve similar performance. Here we have shown that for the same model structure and parameters, stochastic dynamics were robust to parameter variation whereas deterministic dynamics required fine-tuning to produce the experimentally observed phenotype. These results add to a growing body of evidence that stochastic noise can play a beneficial role through the introduction of novel and/or robust functionality, which in turn endows cells with a performance advantage [22],[50]–[52].

Finally, in this first version of our polarisome model we made some compromises to keep the model structure simple. For example, we aggregated the transport and delivery of Spa2 to the membrane along actin cables into a single reaction. We also combined actin cable initiation and polymerization into a single event. We believe that these approximations did not affect the fundamental behaviors exhibited by our model, and we are encouraged that the model was able to reproduce key aspects of the experimental data. Future work will include these additional dynamics in a more complex and detailed model.

## Materials and Methods

### Computational Strategy

We modeled polarization of a yeast cell on a one-dimensional periodic domain (i.e. a circle) representing the membrane, which surrounds a well-mixed cytoplasmic region. On the membrane, the spatial location of the biochemical species was critical to understanding the polarization process. Thus, we tracked the location of populations of proteins on the membrane and allowed them to move via a diffusive random walk. A complete mathematical description of our model is given in the Section 2.3 in Text S1.

Our stochastic model was formalized via the Reaction Diffusion Master Equation (RDME). The inhomogeneous stochastic simulation algorithm (ISSA), a simple and exact simulation method, was used for the parameter space exploration (for a complete description of the RDME and ISSA see Section 4.2 in Text S1). The deterministic model was described by a set of partial differential equations and solved using standard methods (see Section 4.3 in Text S1).

### Tracking

To determine the effectiveness of our model in replicating this phenotype, we performed the following *in silico* experiment in which the initial Cdc42 signal served as an input in one direction (see first panel of Fig. 5B). After 1020 seconds, the input was switched by 180 degrees (see second panel of Fig. 5B). 816 seconds later, we measured how well the polarisome tracked the input signal (see third panel of Fig. 5B, where the polarisome in the simulation has successfully tracked the input signal). Our criterion for successful tracking was that the polarisome be within 90 degrees of the final input (using average location during the final 204 seconds of the simulation). The probability of successful tracking is shown for the stochastic simulation in Fig. S15A in Text S1 and for the deterministic simulation in Fig. S15B in Text S1.

### Full Width at Half-Maximum (FWHM)

We characterized the polarization tightness with the FWHM of a normal distribution fit to the intensity profile data (see Fig. 3C for an example fit to experimental data). The width was measured in the same trajectory as above. The polarisome was allowed to form and stabilize for the first 510 seconds, then the FWHM was sampled at each point in time for the next 510 seconds and the time average was taken to be the width value for that trajectory. Final values presented in the phenotype space plots (Section 7 in Text S1) represent the ensemble mean value.

### Cell Culture and Pheromone Treatment

All yeast strains were derivatives of RJD415 (W303). Genetic techniques were performed according to standard methods. Complete strain details are presented in Table S1 in Text S1. Cells were cultured in YPD (yeast extract-peptone-dextrose) media.

Cells were treated with 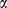-factor for 2 hours before imaging. A low concentration of pheromone (10–20 nM) was used for imaging a dynamic single projection, and a high concentration (100 nM) was used for imaging the second projection formation in the tracking experiments.

### Microscopy

Live yeast cells were immobilized on glass slides with concanavalin A (ConA) in the presence of YPD media containing 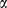-factor. They were then imaged on an Olympus Fluoview confocal microscope with a 60× objective using 488 nm (GFP) and 568 nm (mCherry) excitation wavelengths. Time-lapse images were taken at 30s intervals over a 30 min to 1 hour period. A relatively long dwell time and scan averaging removed much of the imaging noise.

## Supporting Information

Text S1 **Main Supporting Information file.** This file provides more information about (1) the videos, (2) the model, (3) parameter estimation, (4) simulation methodology, (5) imaging and modeling of polarisome dynamics, (6) measuring the polarisome width, (7) the phenotype space plots, and (8) results from the B-model.
(PDF)

Video S1
**Spa2 FRAP experiment** ***in vivo*** **with Spa2-GFP.** One second of video time is equivalent to one second of real time. Spa2-GFP cells treated with 20 nM 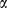-factor for 2 hours were subjected to photobleaching by confocal microscopy, and recovery monitored at ∼1 fps.(MOV)

Video S2 **Spa2 FRAP experiment** ***in silico*** **.** 1s (video time) = 1s (simulation time). The time displayed is such that that the bleaching event occurs at t = 0.
(MOV)

Video S3
**Polarisome tracking experiment** ***in vivo*** **with Ste20-GFP (green) and Spa2-mCherry (red).** 1s (video time) = 30s (real time). Dual-labeled cells containing Ste20-GFP/Spa2-mCherry were treated with 100 nM uniform 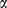-factor for 2 to 3h and imaged under isotropic conditions. Cells undergoing second projection formation were identified and followed at 30s intervals.(MOV)

Video S4 **Polarisome tracking experiment** ***in silico*** **.** 1s (video time) = 30s (simulation time).
(MOV)

Video S5
***spa2*** 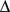 **experiment** ***in vivo*** **with Sec3-GFP.** 1s (video time) = 30s (real time). *spa2* 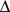 cells containing Sec3-GFP, a polarisome marker, were treated with 20 nM 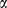-factor for 2h and then imaged under isotropic conditions with frames every 10s.(MOV)

Video S6
***spa2*** 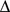 **experiment** ***in silico*** **.** 1s (video time) = 30s (simulation time). The *spa2* 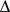 mutant was simulated by setting the value of Spa2_t_ to 10% of the wild-type value (500 molecules/cell vs. 5000 molecules/cell).(MOV)

Video S7
**Dynamic “searching” behavior of Spa2** ***in vivo*** **monitored by Spa2-GFP.** 1s (video time) = 30s (real time). Spa2-GFP cells were treated with 20 nM 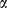-factor for 2h and then imaged under isotropic conditions. The polarisome exhibits dynamic motion at the front of the mating projection. Frames were taken every 10s.(MOV)

Video S8 **Dynamic “searching” behavior of Spa2** ***in silico*** **.** 1s (video time) = 30s (simulation time).
(MOV)

## References

[1] Drubin DG, Nelson WJ (1996) Origins of cell polarity. Cell 84: 335–344. 8608587 10.1016/s0092-8674(00)81278-7

[2] Wedlich-Soldner R, Li R (2003) Spontaneous cell polarization: undermining determinism. Nat Cell Biol 5: 267–270. 12669070 10.1038/ncb0403-267

[3] Arkowitz RA (2001) Cell polarity: Connecting to the cortex. Curr Biol 11: R610–R612. 11516968 10.1016/s0960-9822(01)00365-7

[4] Dohlman HG, Thorner J (2001) Regulation of G protein-initiated signal transduction in yeast: Paradigms and principles. Annual Review of Biochemistry 70: 703–754. 10.1146/annurev.biochem.70.1.703 11395421

[5] Pruyne D, Bretscher A (2000) Polarization of cell growth in yeast: I. Establishment and maintenance of polarity states. J Cell Sci 113: 365–375. 10639324 10.1242/jcs.113.3.365

[6] Layton AT, Savage NS, Howell AS, Carroll SY, Drubin DG, et al (2011) Modeling vesicle trafic reveals unexpected consequences for Cdc42p-mediated polarity establishment. Current Biology 21: 184–194. 21277209 10.1016/j.cub.2011.01.012 PMC3052744

[7] Savage NS, Layton AT, Lew DJ (2012) Mechanistic mathematical model of polarity in yeast. Molecular Biology of the Cell 23: 1998–2013. 22438587 10.1091/mbc.E11-10-0837 PMC3350562

[8] Pruyne D, Bretscher A (2000) Polarization of cell growth in yeast: II. The role of the cortical actin cytoskeleton. J Cell Sci 113: 571–585. 10652251 10.1242/jcs.113.4.571

[9] Alberts B, Bray D, Lewis J, Raff M, Roberts K, et al.. (1994) Molecular Biology of the Cell. New York: Garland Publishing.

[10] Dawes AT, Edelstein-Keshet L (2007) Phosphoinositides and rho proteins spatially regulate actin polymerization to initiate and maintain directed movement in a one-dimensional model of a motile cell. Biophysical Journal 92: 744–768. 17098793 10.1529/biophysj.106.090514 PMC1779977

[11] Iglesias PA, Levchenko A (2002) Modeling the cell's guidance system. Sci STKE 2002: re12. 12209053 10.1126/stke.2002.148.re12

[12] Moore T, Chou CS, Nie Q, Jeon N, Yi TM (2008) Robust spatial sensing of mating pheromone gradients by yeast cells. PLoS One 3: e3865. 19052645 10.1371/journal.pone.0003865 PMC2586657

[13] Evangelista M, Blundell K, Longtine MS, Chow CJ, Adames N, et al (1997) Bni1p, a yeast formin linking Cdc42p and the actin cytoskeleton during polarized morphogenesis. Science 276: 118–122. 9082982 10.1126/science.276.5309.118

[14] Arkowitz R, Lowe N (1997) A small conserved domain in the yeast Spa2p is necessary and sufficient for its polarized location. J Cell Biol 138: 17–36. 9214378 10.1083/jcb.138.1.17 PMC2139937

[15] Madden K, Snyder M (1998) Cell polarity and morphogenesis in budding yeast. Annu Rev Microbiol 52: 687–744. 9891811 10.1146/annurev.micro.52.1.687

[16] Bidlingmaier S, Snyder M (2004) Regulation of polarized growth initiation and termination cycles by the polarisome and Cdc42 regulation. J Cell Biol 164: 207–218. 14734532 10.1083/jcb.200307065 PMC2172334

[17] Dorer R, Boone C, Kimbrough T, Kim J, Hartwell LH (1997) Genetic analysis of default mating behavior in *Saccharomyces cerevisiae* . Genetics 146: 39–55. 9135999 10.1093/genetics/146.1.39 PMC1207953

[18] Meinhardt H (1982) Models of biological pattern formation. London and New York: Academic Press.

[19] Mogilner A, Allard J, Wollman R (2012) Cell polarity: Quantitative modeling as a tool in cell biology. Science 336: 175–179. 22499937 10.1126/science.1216380

[20] Krishnan J, Iglesias P (2004) Uncovering directional sensing: where are we headed? Systems Biology 1: 54–61. 17052115 10.1049/sb:20045001

[21] Ozbudak EM, Becskei A, van Oudenaarden A (2005) A system of counteracting feedback loops regulates Cdc42p activity during spontaneous cell polarization. Developmental Cell 9: 565–571. 16198298 10.1016/j.devcel.2005.08.014

[22] Arkin A, Ross J, McAdams HH (1998) Stochastic kinetic analysis of developmental pathway bifurcation in phage λ-infected *Escherichia coli* cells. Genetics 149: 1633–1648. 9691025 10.1093/genetics/149.4.1633 PMC1460268

[23] McAdams H, Arkin A (1997) Stochastic mechanisms in gene expression. Proc National Academy Sciences USA 94: 814–819. 10.1073/pnas.94.3.814 PMC19596 9023339

[24] Maheshri N, O'Shea E (2007) Living with noisy genes: How cells function reliably with inherent variability in gene expression. Annu Rev Biophys Biomolec Struct 36: 413–434. 10.1146/annurev.biophys.36.040306.132705 17477840

[25] Houchmandzadeh B, Wieschaus E, Leibler S (2002) Establishment of developmental precision and proportions in the early drosophila embryo. Nature 415: 798–802. 11845210 10.1038/415798a

[26] Deco G, Rolls E, Romo R (2009) Stochastic dynamics as a principle of brain function. Progress in Neurobiology 88: 1–16. 19428958 10.1016/j.pneurobio.2009.01.006

[27] Rao C, Wolf D, Arkin A (2002) Control, exploitation, and tolerance of intracellular noise. Nature 420: 231–237. 12432408 10.1038/nature01258

[28] Wang X, Hao N, Dohlman HG, Elston TC (2006) Bistability, stochasticity, and oscillations in the mitogen-activated protein kinase cascade. Biophysical Journal 90: 1961–1978. 16361346 10.1529/biophysj.105.073874 PMC1386776

[29] Gillespie DT (2000) The chemical Langevin equation. The Journal of Chemical Physics 113: 297–306.

[30] Fange D, Elf J (2006) Noise-induced Min phenotypes in *E. coli* . PLoS Comput Biol 2: e80. 16846247 10.1371/journal.pcbi.0020080 PMC1484588

[31] Wedlich-Soldner R, Altschuler S, Wu L, Li R (2003) Spontaneous cell polarization through actomyosin-based delivery of the Cdc42 GTPase. Science 299: 1231–1235. 12560471 10.1126/science.1080944

[32] Altschuler SJ, Angenent SB, Wang Y, Wu LF (2008) On the spontaneous emergence of cell polarity. Nature 454: 886–889. 18704086 10.1038/nature07119 PMC2562338

[33] Marco E, Wedlich-Soldner R, Li R, Altschuler S, Wu L (2007) Endocytosis optimizes the dynamic localization of membrane proteins that regulate cortical polarity. Cell 129: 411–422. 17448998 10.1016/j.cell.2007.02.043 PMC2000346

[34] Fujiwara T, Tanaka K, Mino A, Kikyo M, Takahashi K, et al (1998) Rho1p-Bni1p-Spa2p interactions: implication in localization of Bni1p at the bud site and regulation of the actin cytoskeleton in *Saccharomyces cerevisiae* . Mol Biol Cell 9: 12211233. 10.1091/mbc.9.5.1221 PMC25343 9571251

[35] Sanft K, Gillespie D, Petzold L (2011) Legitimacy of the stochastic michaelis??menten approximation. Systems Biology, IET 5: 58–69. 10.1049/iet-syb.2009.0057 21261403

[36] Yu H, Wedlich-Soldner R (2011) Cortical actin dynamics: Generating randomness by formin(g) and moving. BioArchitecture 1: 165–168. 22069508 10.4161/bioa.1.4.17314 PMC3210520

[37] Goode BL, Eck MJ (2007) Mechanism and function of formins in the control of actin assembly. Annual Review of Biochemistry 76: 593–627. 10.1146/annurev.biochem.75.103004.142647 17373907

[38] Liu H, Bretscher A (1992) Characterization of TPM1 disrupted yeast cells indicates an involvement of tropomyosin in directed vesicular transport. J Cell Biol 118: 285–299. 1629236 10.1083/jcb.118.2.285 PMC2290051

[39] Valdez-Taubas J, Pelham HR (2003) Slow diffusion of proteins in the yeast plasma membrane allows polarity to be maintained by endocytic cycling. Current Biology 13: 1636–1640. 13678596 10.1016/j.cub.2003.09.001

[40] Sprague BL, Pego RL, Stavreva DA, McNally JG (2004) Analysis of binding reactions by fluorescence recovery after photobleaching. Biophysical Journal 86: 3473–3495. 15189848 10.1529/biophysj.103.026765 PMC1304253

[41] Evengelista M, Pruyne D, Amberg DC, Boone C, Bretscher A (2002) Formins direct Arp2/3-independent actin filament assembly to polarize cell growth in yeast. Nat Cell Biol 4: 32–41. 11740490 10.1038/ncb718

[42] Carlier MF, Laurent V, Santolini J, Melki R, Didry D, et al (1997) Actin Depolymerizing Factor (ADF/Cofilin) Enhances the Rate of Filament Turnover: Implication in Actin-based Motility. TheJournal of Cell Biology 136: 1307–1322. 9087445 10.1083/jcb.136.6.1307 PMC2132522

[43] Paliwal S, Iglesias P, Campbell K, Hilioti Z, Groisman A, et al (2007) MAPK-mediated bimodal gene expression and adaptive gradient sensing in yeast. Nature 446: 46–51. 17310144 10.1038/nature05561

[44] Tanaka H, Yi TM (2009) Synthetic morphology using alternative inputs. PLoS ONE 4: e6946. 19746161 10.1371/journal.pone.0006946 PMC2735001

[45] Chou CS, Nie Q, Yi TM (2008) Modeling robustness tradeoffs in yeast cell polarization induced by spatial gradients. PLoS One 3: e3103. 21267054 10.1371/journal.pone.0003103 PMC3021495

[46] Meinhardt H (1999) Orientation of chemotactic cells and growth cones: models and mechanisms. J Cell Sci 112: 2867–2874. 10444381 10.1242/jcs.112.17.2867

[47] Yu J, Crevenna A, Bettenbuhl M, Freisinger T, Wedlich-Soldner R (2011) Cortical actin dynamics driven by formins and myosin V. J Cell Sci. 124: 1533–1541. 10.1242/jcs.079038 21486946

[48] Barkai N, Leibler S (1997) Robustness in simple biochemical networks. Nature 387: 913–7. 9202124 10.1038/43199

[49] Yi TM, Huang Y, Simon MI, Doyle J (2000) Robust perfect adaptation in bacterial chemotaxis through integral feedback control. Proc Natl Acad Sci USA 97: 4649–4653. 10781070 10.1073/pnas.97.9.4649 PMC18287

[50] Paulsson J, Berg OG, Ehrenberg M (2000) Stochastic focusing: Fluctuation-enhanced sensitivity of intracellular regulation. Proc Natl Acad Sci USA 97: 7148–7153. 10852944 10.1073/pnas.110057697 PMC16514

[51] Vilar JMG, Kueh HY, Barkai N, Leibler S (2002) Mechanisms of noise-resistance in genetic oscillators. Proc Natl Acad Sci USA 99: 5988–5992. 11972055 10.1073/pnas.092133899 PMC122889

[52] Weinberger LS, Burnett JC, Toettcher JE, Arkin AP, Schaffer DV (2005) Stochastic gene expression in a lentiviral positive-feedback loop: HIV-1 Tat fluctuations drive phenotypic diversity. Cell 122: 169–182. 16051143 10.1016/j.cell.2005.06.006

