# Supplementary material for: Spatial Stochastic Dynamics Enable Robust Cell Polarization
Source: PLoS Comput Biol. 2013 Jul 25;9(7):e1003139. doi: 10.1371/journal.pcbi.1003139 (PMC3723497; doi:10.1371/journal.pcbi.1003139)
Supplement: Text S1 — Main Supporting Information file. This file provides more information about (1) the videos, (2) the model, (3) parameter estimation, (4) simulation methodology, (5) imaging and modeling of polarisome dynamics, (6) measuring the polarisome width, (7) the phenotype space plots, and (8) results from the B-model. (PDF) [file pcbi.1003139.s001.pdf]

Text S1
Supporting Information
for
Stochastic Spatial Dynamics Enable Robust Cell Polarization

June 13, 2013

Contents

1 Videos **2**

2 Polarisome **3**
- 2.1 Strain Details . . . 3
- 2.2 Polarisome Model Background . . . 3
- 2.3 Model of Polarisome Formation in Budding Yeast . . . 4

3 Parameter Estimation **7**
- 3.1 Model of FRAP . . . 7
- 3.2 FRAP simulation . . . 8
- 3.3 FRAP experiments and Latrunculin A . . . 8
- 3.4 B_{off} and S_{off} . . . 12
- 3.5 B_{on} and S_{on} . . . 12
- 3.6 A_{on} . . . 13
- 3.7 A_{off} . . . 14
- 3.8 Calculation of D . . . 15

4 Simulation of the Polarisome model **16**
- 4.1 Stochastic Simulation Algorithm . . . 16
- 4.2 Inhomogeneous Stochastic Simulation Algorithm (ISSA) . . . 17
- 4.3 Spatial Deterministic Simulation Methods . . . 17

5 Experimental Images and Modeling of Polarisome Dynamics **18**
- 5.1 Myo2-Spa2 colocalization during the pheromone response . . . 18
- 5.2 Reporters of active Cdc42 . . . 18
- 5.3 Polarisome Searching and Tracking Phenotypes . . . 19
- 5.4 Tracking by the polarisome under gradient conditions . . . 19
- 5.5 Tracking overlap time . . . 19

6 Measuring the Polarisome Width **25**

7 Phenotype Space Plots **26**

8 Simulation results for B-Model **28**

1 Videos

All simulations videos use the S-model with the nominal parameter set ($K_\mathrm{m} = 3500$, $B_\mathrm{fb}/B_\mathrm{on}$ratio $=$ 7.5).

FRAP. In videos S1 and S2 we show experiment and simulation videos of a FRAP experiment where a constant width of the cell membrane is exposed to intense laser light, and the fluorescently tagged proteins in that region are bleached (become non-photoactive). The dynamics of diffusion and cytoplasmic recycling allow for new fluorescently tagged proteins to enter that region and thus fluorescence returns to the bleached region (recovery).

Video S1: Spa2 FRAP experiment *in vivo* with Spa2-GFP. One second of video time is equivalent to one second of real time. Spa2-GFP cells treated with 20 nM α-factor for 2 hours were subjected to photobleaching by confocal microscopy, and recovery monitored at $\sim$1 fps.

Video S2: Spa2 FRAP experiment *in silico.* 1s (video time) = 1s (simulation time). The time displayed is such that that the bleaching event occurs at t=0.

Tracking. In videos S3 and S4 we show experiment and simulation videos of the tracking process of the polarisome (red) following the Cdc42a polarization (green). Initially both Spa2 and Cdc42a are polarized to the right. A short while later the Cdc42a polarization switches to the left side, but the polarisome remains on the right, and then a short time later the polarisome follows the Cdc42a polarization to the left side.

Video S3: Polarisome tracking experiment *in vivo* with Ste20-GFP (green) and Spa2-mCherry (red). 1s (video time) = 30s (real time). Dual-labeled cells containing Ste20-GFP/Spa2-mCherry were treated with 100 nM uniform α-factor for 2 to 3 h and imaged under isotropic conditions. Cells undergoing second projection formation were identified and followed at 30 s intervals.

Video S4: Polarisome tracking experiment *in silico.* 1s (video time) = 30s (simulation time).

***spa2*Δ**. In videos S5 and S6 we show experiment and simulation videos of the multi-polarisome phenotype of the *spa2*Δ mutants. The multiple polarisome foci exhibit a more noisy and transient polarization compared to the wild-type.

Video S5: *spa2*Δ experiment *in vivo* with Sec3-GFP. 1s (video time) = 30s (real time). *spa2*Δ cells containing Sec3-GFP, a polarisome marker, were treated with 20 nM α-factor for 2 h and then imaged under isotropic conditions with frames every 10s.

Video S6: *spa2*Δ experiment *in silico.* 1s (video time) = 30s (simulation time). The *spa2*Δ mutant was simulated by setting the value of $\mathrm{Spa2_t}$ to 10% of the wild-type value (500 molecules/cell vs. 5000 molecules/cell).

Searching. In videos S7 and S8 we show experiment and simulation videos of the dynamic "searching" behavior of the polarisome. The polarisome (red) localizes to the front of the Cdc42a polarized region (green), but is not static in its location within this region.

Video S7: Dynamic "searching" behavior of Spa2 *in vivo* monitored by Spa2-GFP. 1s (video time) = 30s (real time). Spa2-GFP cells were treated with 20 nM α-factor for 2 h and then imaged under isotropic conditions. The polarisome exhibits dynamic motion at the front of the mating projection. Frames were taken every 10s.

Video S8: Dynamic "searching" behavior of Spa2 *in silico.* 1s (video time) = 30s (simulation time).

2 Polarisome

2.1 Strain Details

Strain	Genotype	Source
RJD415	*MATa can1-100 leu2-3-112 his3-11-15 trp1-1 ura3-1 ade2-1 pep4*Δ*::TRP1 bar1*Δ*::LEU2*	Ray Deshaies
TMY065	RJD415 *sec3::SEC3-GFP-HIS5*	This study
TMY465	RJD415 *sec3::SEC3-GFP-HIS5 spa2*Δ*::KAN*^{R}	This study
TMY466	RJD415 *sec3::SEC3-GFP-HIS5 spa2::SPA2-mCherry-KAN*^{R}	This study
TMY523	RJD415 *ste20::STE20-GFP-HIS5 spa2::SPA2-mCherry-KAN*^{R}	This study
TMY524	RJD415 *bni1::BNI1-GFP-HIS5 spa2*Δ*::KAN*^{R}	This study
TMY525	RJD415 *bni1::BNI1-GFP-HIS5 spa2::SPA2-mCherry-KAN*^{R}	This study
TMY526	RJD415 *gic2::GIC2-208*Δ*-GFP-HIS5 spa2::SPA2-mCherry-KAN*^{R}	This study
TMY527	RJD415 *myo2::MYO2-GFP-HIS5 spa2::SPA2-mCherry-KAN*^{R}	This study

Table S1: Yeast strains used in this study.

2.2 Polarisome Model Background

Sheu et al. [1] first isolated the yeast polarisome as a 12S particle by velocity sedimentation. The three proteins Spa2, Pea2, and Bud6 were shown to be components of this structure. Subsequent work examining protein-protein interactions of the above has identified the formin Bni1 and the scaffold protein Sph1 as additional components of the polarisome [2]. Functionally, the polarisome is involved in the assembly of polarized actin structures, cables and patches, during budding and mating. In addition, the polarisome marks a site of exocytosis where vesicles are transported to the cell membrane or secreted. Indeed, the polarisome is closely associated with the exocyst which is the docking site for secretory vesicles. The key polarisome protein is Bni1, which initiates the polymerization of actin cables along which vesicles move using myosin motors.

The polarisome has been identified in other filamentous fungi such as *Candida albicans* and *Ashbya gossypii*. Interestingly, in both *Candida* and *Ashbya* there is an auxiliary structure called the Spitzenkörper that forms at an intermediate location near the polarization tip where vesicles accumulate before they are ultimately transported to the polarisome and exocyst [3, 4]. This structure has not been characterized yet in *S. cerevisiae*. Here we adopt a simplified view in which vesicles are directly transported to the plasma membrane from the cytoplasm. Thus, intermediate membrane structures are important but the ultimate distribution on the plasma membrane depends on the actin cytoskeleton which we explicitly model.

In this work we represent the plasma membrane as a static structure. One concern raised by Layton et al. [5] is that exocytic vesicles not only deliver protein to the membrane (e.g. Spa2), but also lipid which can dilute the concentration of membrane proteins. However, on the fast time-scale of our model (seconds), we assume that lipid exocytosis is matched by lipid endocytosis, resulting in pseudo steady-state lipid dynamics. More importantly, the lipid and protein dynamics are uncoupled in terms of endocytosis. The polarisome proteins Bni1 and Spa2 are not associated with endocytic

vesicles [6], and in our model they dissociate from the membrane. Thus, while the level of lipid is constant, the amount of membrane protein can increase or decrease. We note that for other proteins on longer time-scales, the argument of Layton et al. may be more relevant.

2.3 Model of Polarisome Formation in Budding Yeast

The model incorporates the reactions depicted in Fig. 2 (main text) representing the core polarisome spatial dynamics. The first component is Cdc42a (active Cdc42) on the membrane, which has a spatially-varying concentration but is constant in time; Cdc42a is the input to the system. The second component, Bni1, is initially homogeneously distributed in the cytoplasm. It is recruited to the membrane by Cdc42a [7] at a rate proportional to the concentration of Cdc42a at that location. Once on the membrane, Bni1 can diffuse laterally or return to the cytoplasm (Fig. 2A). Bni1 is a formin [7, 6], thus on the membrane it nucleates new actin cables (Fig. 2B), as well as acting to stabilize existing actin cables (similar to the generalized mechanisms described in [8]). We assume a finite number of actin cables, thus in polarisome formation there will be a competition for this resource. For simplicity, we model only actin attaching to (A_{on}) and detaching from (A_{off}) the membrane, not the full polymerization process. The fourth component, Spa2, is delivered to the membrane by myosin-mediated transport along actin cables (Fig. 2C). Spa2 can diffuse on the membrane or return to the cytoplasm (S_{off}). The positive feedback loop is completed by membrane-associated Spa2 recruiting Bni1 from the cytoplasm to the membrane, and by Spa2-mediated inhibition of actin depolymerization (Fig. 2D). Technically, Spa2 inhibiting actin depolymerization is not a model assumption built into our model structure, but instead is a biological hypothesis representing the region of parameter space in which $K_m < \infty$. We also explore the possibility that the polarisome component does not inhibit actin depolymerization ($K_m = \infty$). We model the chemical reactions primarily by simple mass action kinetics, resulting in a model containing the input, 3 species which exist in cytoplasmic and membrane forms, and 8 key parameters. The only non-mass action term in the model is an inhibition term taking the form of a Michaelis-Menten type expression to describe actin cable stabilization. Recent work [9] has shown that the Michaelis-Menten approximation is valid for stochastic models, under the same conditions for which it is valid for deterministic models. We have checked to ensure that our model satisfies those conditions. See Tables S2, S3, and S4 for a complete list of the species, reactions, and parameters for the model.

The association of Bni1 with the membrane via interaction with Cdc42a can be thought of as forming a complex between the two. In this interpretation, Bni1_{m} represents the Bni1_{m}-Cdc42a complex. Subsequent dissociation can occur via a reversible or irreversible mechanism. We believe that irreversible internalization of Bni1 from the membrane is the dominant mechanism because once Bni1 initiates actin cable assembly and interacts with the resulting actin filaments, the exact reverse reaction is no longer possible. Indeed there is solid biological evidence that Bni1 is internalized by a mechanism different from membrane association. Most notably, we and others [6] have data that show that Bni1 internalization depends on actin, whereas association with the membrane does not.

We did not represent bound complexes in the model; explicitly representing the complexes would increase the number of species in the model resulting in computationally more expensive simulations without significantly affecting the dynamics. Each of the membrane species was in large excess over the species being recruited to the membrane. Near the polarization peak there are greater than 200 molecules of Cdc42a and less than 10 molecules of Bni1; expected Spa2 population on the membrane is 22.5 times that of Bni1; the expected membrane population of Bni1 in wild-type cells is 10 times

higher than the expected membrane population of actin cables. As a result, saturation of the recruiting membrane species was not an issue. The association of Bni1 with the membrane via Cdc42a is by forming a complex. Thus, Bni1m represents the Bni1m-Cdc42a complex.

**Species**	**Description**
$Bni1_{\mathrm{c}}$	Bni1 in the cytoplasm
$Bni1_{\mathrm{m}}$	Bni1 on the plasma membrane
$Spa2_{\mathrm{c}}$	Spa2 in the cytoplasm
$Spa2_{\mathrm{m}}$	Spa2 on the plasma membrane
$Actin_{\mathrm{c}}$	Unpolymerized actin in the cytoplasm
$Actin_{\mathrm{m}}$	Polymerized actin on the plasma membrane

Table S2: Biochemical species for the polarisome model.

Initial Conditions and Total Molecule Numbers For the initial conditions, all molecules of Bni1, Spa2, and actin were in the cytoplasm. The spatial profile of Cdc42a was given by a Gaussian with $\sigma = 20.3$ (corresponding to a FWHM of 48°) and a maximum value of 238. Using the estimated total amount of Cdc42a, we fit the maximum so that the Gaussian would integrate to the total value.

The total numbers of Bni1 and Spa2 molecules were estimated from fluorescence intensity measurements of Bni1-GFP and Spa2-GFP compared to levels of Ste2-GFP on the membrane (10,000 molecules/cell) [12]. Using simulations, we explored a range of total actin cable numbers from as low as 10 to over 200. The work of Yu et al. [13] estimated that there were approximately 7 actin cables on the membrane in a growing bud; this number is likely to be a lower bound because of the difficulty counting cables. We found that simulations with 40 total actin cables (which corresponds to 20 cables on the membrane) accurately reproduced the spatio-temporal characteristics of the polarisome.

Model Cell Geometry The model was implemented on a 1-D periodic domain (circular membrane) of diameter 8 μm. This domain was discretized into 160 equally sized voxels of circumferential length $\frac{\pi}{20}$ μm. We assumed that protein in the cytoplasm diffuses much faster than on the membrane, thus we treated the cytoplasm as well mixed. Therefore it was assumed that each voxel on the membrane had equal access to the same cytoplasmic pool of protein (similar assumptions were made in [14, 15], and is consistent with the results from [16]).

Stochastic simulations are expressed in terms of molecule populations (discrete integer values) while deterministic simulations are expressed in terms of concentrations (continuous values), thus a conversion of reaction rate constants is required to convert the reaction network from one formulation to the other. In our model, we did this by normalizing the reaction rate constants with respect to the voxel sizes, thus the values of the reaction rate constants are the same in both formulations.

Reaction	Reaction Rate	Description/References
$Bni1_{\mathrm{c}} + Cdc42_{\mathrm{m}} \rightarrow Bni1_{\mathrm{m}} + Cdc42_{\mathrm{m}}$	$B_{\mathrm{on}} \times [Bni1_{\mathrm{c}}] \times [Cdc42_{\mathrm{m}}]$	Active Cdc42 recruits Bni1 to the membrane [7]
$Bni1_{\mathrm{m}} \rightarrow Bni1_{\mathrm{c}}$	$B_{\mathrm{off}} \times [Bni1_{\mathrm{m}}]$	Bni1 dissociates from the membrane via actin patches [6]
$Actin_{\mathrm{c}} + Bni1_{\mathrm{m}} \rightarrow Actin_{\mathrm{m}} + Bni1_{\mathrm{m}}$	$A_{\mathrm{on}} \times [Bni1_{\mathrm{m}}] \times [Actin_{\mathrm{c}}]$	Bni1 initiates polymerization of actin cables on the membrane [10]
$Actin_{\mathrm{m}} + Spa2_{\mathrm{m}} \rightarrow Actin_{\mathrm{c}} + Spa2_{\mathrm{m}}$	$A_{\mathrm{off}} \frac{K_{\mathrm{m}}}{K_{\mathrm{m}} + [Spa2_{\mathrm{m}}]} \times [Actin_{\mathrm{m}}]$	Spa2 inhibits actin depolymerization
$Spa2_{\mathrm{c}} + Actin_{\mathrm{m}} \rightarrow Spa2_{\mathrm{m}} + Actin_{\mathrm{m}}$	$S_{\mathrm{on}} \times [Spa2_{\mathrm{c}}] \times [Actin_{\mathrm{m}}]$	Spa2 is transported along actin cables to the membrane [11]
$Spa2_{\mathrm{m}} \rightarrow Spa2_{\mathrm{c}}$	$S_{\mathrm{off}} \times [Spa2_{\mathrm{m}}]$	Spa2 dissociates from the membrane
$Bni1_{\mathrm{c}} + Spa2_{\mathrm{m}} \rightarrow Bni1_{\mathrm{m}} + Spa2_{\mathrm{m}}$	$B_{\mathrm{fb}} \times [Bni1_{\mathrm{c}}] \times [Spa2_{\mathrm{m}}]$	Spa2 on the membrane recruits Bni1 to the membrane [11]

Table S3: Reactions and reaction rates for the polarisome model. The reaction rates are all first-order or second-order (bilinear) kinetics except for the actin depolymerization reaction which contains an inhibition term.

Parameter	Value
B_{on}	$1.6 \times 10^{-6}\ mol^{-1}s^{-1}$
B_{off}	$0.25\ s^{-1}$
B_{fb}	$1.9 \times 10^{-5}\ mol^{-1}s^{-1}$
A_{on}	$7.7 \times 10^{-5}\ mol^{-1}s^{-1}$
A_{off}	$0.018\ s^{-1}$
K_{m}	$3500\ mol$
S_{on}	$0.16\ mol^{-1}s^{-1}$
S_{off}	$0.35\ s^{-1}$
$Bni1_{\mathrm{t}}$	1000 molecules
$Spa2_{\mathrm{t}}$	5000 molecules
$Actin_{\mathrm{t}}$	40 molecules
D	$0.0053\ \mu\mathrm{m}\ s^{-2}$

Table S4: Parameters and parameter values for the polarisome model. In Section 3 we outline the estimation procedure for D, B_{off}, S_{off}, A_{on}, S_{on} and S_{off}. As described in the main text, this leaves two free parameters: the $B_{\mathrm{fb}}/B_{\mathrm{on}}$ ratio and the (K_{m}, A_{off}) relationship (derivation in Section S3). These two parameters were selected as outlined in the main text. The simulations shown in Fig. 2 (main text) use these parameter values.

3 Parameter Estimation

3.1 Model of FRAP

There are two key components to fluorescence recovery on the membrane: diffusion along the membrane and the cycling rate of proteins on and off the membrane. It is clear that the recovery due to diffusion is dependent only on the diffusion coefficient D. Less clear is which rate is important for recovery due to cycling.

Imagine a system in which diffusion is extremely slow (essentially $D = 0$). In this case, recovery would be due only to delivering protein to the membrane. Due to the insignificance of diffusion, we can think of this system in terms of ordinary differential equations (ODEs). There are four relevant populations: x_c is the cytoplasmic protein population, x_{m1} is the membrane protein population in the regions that will NOT be bleached, x_{m2} is the membrane protein population in the regions that will be bleached and x_{mb} is the bleached membrane population. The full set of ODEs to describe this system are given by Equation 1, with k_{on} as the delivery rate to the membrane, k_{off} the rate that proteins leave the membrane and $0 \leq REG_{m1} \leq 1$ is the fraction of the membrane that will NOT be bleached:

$$\begin{aligned}
\dot{x}_c &= -k_{on} \times x_c + k_{off}(x_{m1} + x_{m2} + x_{mb}), \\
\dot{x}_{m1} &= k_{on} \times (REG_{m1}) \times x_c - k_{off} x_{m1}, \\
\dot{x}_{m2} &= k_{on} \times (1 - REG_{m1}) \times x_c - k_{off} x_{m2}, \\
\dot{x}_{mb} &= -k_{off} x_{mb}.
\end{aligned} \tag{1}$$

The total protein population in the system is C. From the perspective of the cytoplasm, there is only one membrane population: $x_m = x_{m1} + x_{m2} + x_{mb} = C - x_c$, therefore the steady state of the cytoplasmic pool is given by $x_{css} = \frac{k_{off}}{k_{on}+k_{off}} C$. For the membrane populations the steady state values are $x_{m1ss} = \frac{k_{on}}{k_{on}+k_{off}} C \times REG_{m1}$, $x_{m2ss} = \frac{k_{on}}{k_{on}+k_{off}} C \times (1 - REG_{m1})$ and $x_{mbss} = 0$.

In our FRAP experiments we bleach the system once it is in steady state. To perform bleaching computationally we simply solve Equation 1 with the following initial condition:

$$\begin{aligned}
x_c(0) &= x_{css}, \\
x_{m1}(0) &= x_{m1ss}, \\
x_{m2}(0) &= 0, \\
x_{mb}(0) &= x_{m2ss}.
\end{aligned} \tag{2}$$

Note that the total membrane population is still equal to the steady state membrane population, thus the total membrane population x_m and the cytoplasmic population x_c will NOT change. The dynamics of the bleached region x_{m2} is governed by:

$$\dot{x}_{m2} = k_{on} \times (1 - REG_{m1}) \times x_c - k_{off} x_{m2} = k_{on} \times (1 - REG_{m1}) \times \frac{k_{off}}{k_{on} + k_{off}} C - k_{off} x_{m2}. \tag{3}$$

Equation 3 is an inhomogeneous linear system, thus is straightforward to solve analytically, with the intial condition $x_{m2}(0) = 0$, yielding

$$x_{m2}(t) = \frac{k_{off} \times k_{on}}{k_{off}(k_{on} + k_{off})} C \times (1 - REG_{m1})(1 - \exp^{-k_{off}t}). \tag{4}$$

The quantitative measure used in FRAP is the time to half full recovery. From Equation 4 it is clear that the max value is $x_{m2ss} = \frac{k_{on}}{(k_{on}+k_{off})} C \times (1 - REG_{m1})$. Therefore, the time to half recovery is:

$$t_{max/2} = \frac{\log(2)}{k_{off}}. \tag{5}$$

Intuitively, the rate of recovery due to protein delivery would depend on the rate of protein transport to the membrane, however Equation 5 gives the counterintuitive conclusion that this component of recovery is dependent entirely on k_{off}.

3.2 FRAP simulation

Equation 5 above provides the expected contribution to recovery from protein cycling on and off the membrane. The implication is that if FRAP were performed in simulation, the Spa2 and Bni1 recovery times should vary with diffusion coefficient and off rate, but not with on rate.

To test this we performed FRAP on the full stochastic model presented in this paper, across a range of S_{off} and B_{off} parameters (rates at which Spa2 and Bni1 leave the membrane) and D. The results, as can be seen in Figs. S1 and S2, are consistent with the theory. In the top row of Figs. S1 and S2 we see similar changes in the time to half recovery with variation in D and S_{off} or B_{off}. However, in the bottom row of these Figures, we see that as we vary S_{on} and B_{on} (rate at which Spa2 and Bni1 are transported to the membrane), the time to half full recovery stays fairly constant across 3 orders of magnitude variation, while it varies in the same way as the top row across D. Finally, note that as D goes to zero in the top middle panel, the time to half recovery goes to what Equation 5 predicts for diffusion free recovery (black dashed line). Similarly, as S_{off} or B_{off} goes to zero in the top right panel, the time to half recovery goes to what Equation 15 predicts for pure diffusion recovery (black dashed line). Proteins that cycle on and off the membrane present a challenge to determining membrane diffusion coefficients directly with FRAP. However, if we are able to determine the diffusion coefficient on the membrane independent of the off rate, the above implies that we could then calculate an off rate from FRAP data on WT cells.

3.3 FRAP experiments and Latrunculin A

Treating yeast cells with Latrunculin A (LatA) results in the rapid depolymerization of actin polymers. One consequence of this is that actin cables disassemble and very quickly the membrane population of Spa2 disappears. This is consistent with our model in a very intuitive way: if you knock out the delivery system for Spa2 to the membrane but maintain its ability to fall off the membrane, you see a rapid decline in the membrane population. Unfortunately, LatA does not induce instantaneous depolymerization, so we cannot use it to directly measure the off rate for Spa2.

However, another advantage of treatment with LatA is that the Bni1 population reaches a higher stable membrane population. This is due to the loss of actin structures, which facilitate internalization of Bni1 from the membrane [6]. In passing, we note that we model the Bni1 off-reaction from the membrane as a first-order process that does not depend on actin cables. This is a reasonable assumption because we do not model actin patches in our current model, which may be

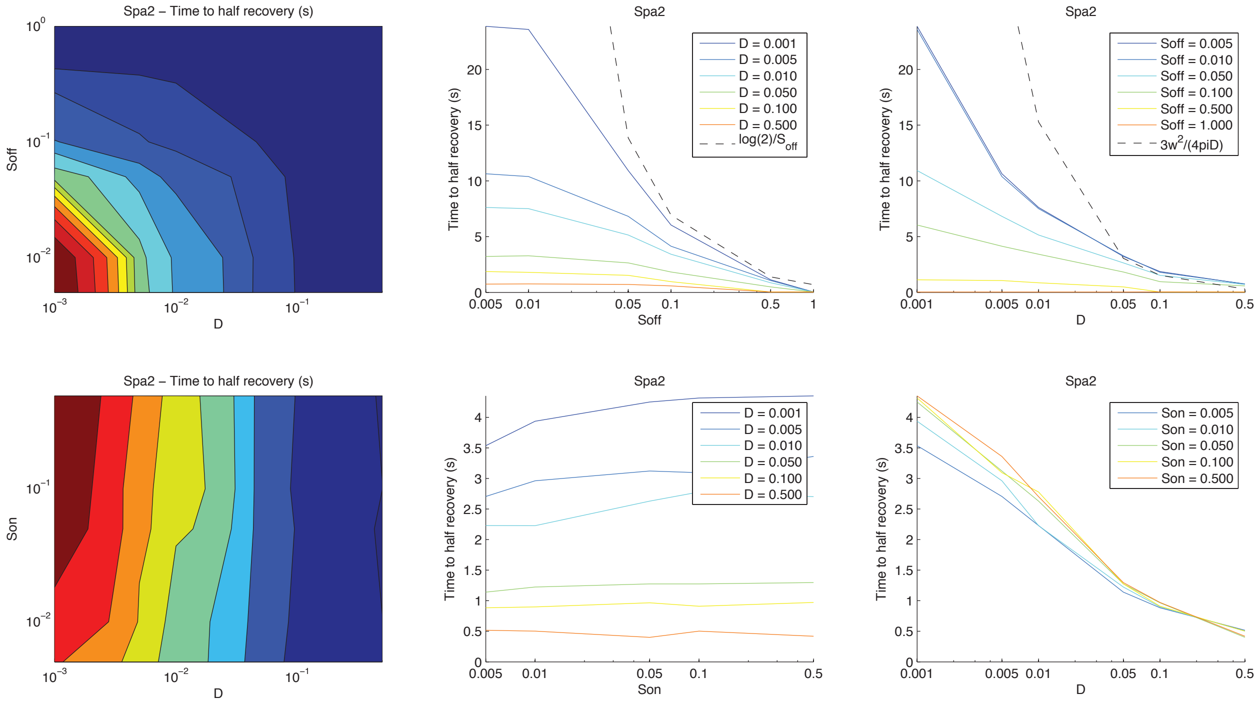

Figure S1: Top: The first panel shows the topology of variation in time to half recovery across various S$_{off}$ (vertical axis) and D (horizontal axis) values (both axis in log scale). The second and third panels show the same data with constant D and varying S_{off} (middle) and constant S_{off} with varying D (right) (horizontal axis in log scale). Note that as D decreases, the curves approach the theory for recovery time for the diffusion free case (dashed black line). Similarly, as S_{off} decreases the curves approach the theory for recovery time for pure diffusion (dashed black line). $S_{on} = 0.32mol^{-1}s^{-1}$. **Bottom:** The panels in the second row follow the same progression as in the top row, except that now S_{on} is being varied in place of S_{off}. From these plots it is clear that varying S_{off} and D causes a change in recovery time, but changing S_{on} has no effect on recovery times. $S_{off} = 0.35s^{-1}$

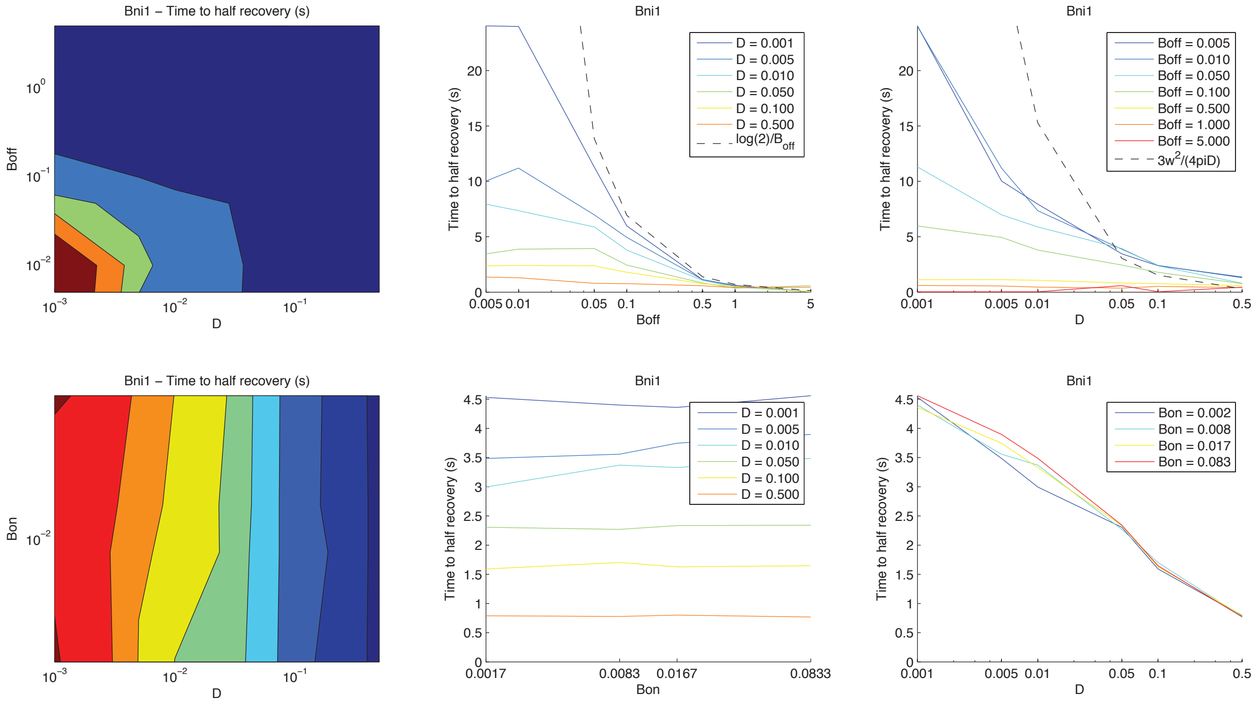

Figure S2: The layout is similar to that of Figure S1. Here, B_{off} and B_{on} are varied, and time to half recovery for Bni1 is reported. As with Spa2, we find that varying the on rate does not affect the time to half recovery, but B_{off} and D both cause a change in the recovery time. Again, note that as D decreases, the curves approach the theory for recovery time for the diffusion free case (dashed black line). Similarly, as B_{off} decreases the curves approach the theory for recovery time for pure diffusion (dashed black line). **Top:** $B_{on} = 0.25 mol^{-1} s^{-1}$. **Bottom:** $B_{off} = 0.4 s^{-1}$.

involved in Bni1 internalization Further, we point out that although actin-dependent internalization is not explicitly modeled, we have implicitly incorporated this process into the rate constant of Bni1 dissociation from the membrane. In the future we plan to model actin-dependent endocytosis more explicitly. The implication of the stable population without removal is that the Bni1 proteins persist on the membrane. Therefore, if we treat cells with LatA, wait for the Spa2 to fall off and then perform FRAP on the Bni1 population we can determine the diffusion coefficient of Bni1 directly. We performed this experiment, and the results are in the bottom panel of Fig. S3. The recovery time to half max for Bni1 in a FRAP experiment with LatA is 26s (see the bottom panel of Fig. S3). In the experiments (and simulations), a region of 0.8 μm was bleached. Therefore, by Equation 15 in Section 3.8 we can infer a diffusion coefficient of $D = 0.0053\frac{\mu m^2}{s}$.

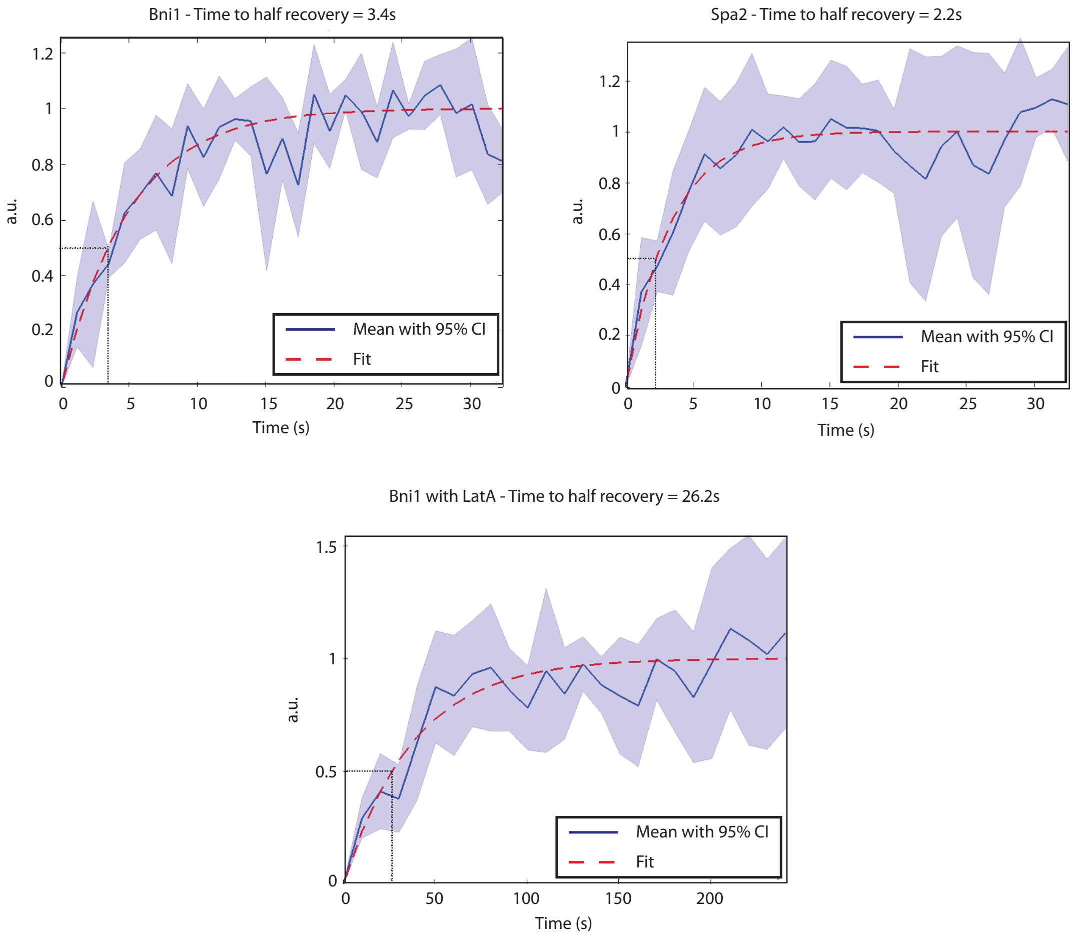

Figure S3: All three plots represent the average of 5 FRAP experiments. The fit curve is used to determine the time to half-recovery, therefore the data is normalized by the maximum value of the fit curve. **Top left:** FRAP recovery curve for Bni1 in the WT. **Top right:** FRAP recovery curve for Spa2 in the WT. **Bottom:** FRAP recovery curve for Bni1 in LatA treated cells.

3.4 $\mathbf{B_{off}}$ and $\mathbf{S_{off}}$

Given the diffusion coefficient for Bni1, we can use Fig. S2 to calculate B_{off}. We find $B_{off} \approx 0.25s^{-1}$. Spa2 and Bni1 are similar sized proteins, neither of which have a transmembrane domain. Therefore, it is reasonable to assume that they have a similar diffusion coefficient. Using this D, we can calculate $S_{off} \approx 0.35s^{-1}$.

3.5 $\mathbf{B_{on}}$ and $\mathbf{S_{on}}$

We solve for the steady state for total membrane fraction of the protein. This fraction is dependent completely on the on and off rates:

$$\dot{x}_m = -k_{off}x_m + k_{on}x_c = -k_{off}x_m + k_{on}(C - x_m) \Rightarrow \frac{x_{mss}}{C} = \frac{k_{on}}{k_{off} + k_{on}}. \tag{6}$$

We already have the off rates from Section 3.4. To find the on rates for Bni1 (B_{on}) and Spa2 (S_{on}) we need to know the steady state fraction of each of these species, as well as the steady state fraction of actin cables, on the membrane. From whole cell imaging (see Fig. S4) we know that ~90% of Spa2 (0.9×5000 molecules = 4500 molecules) and ~20% of Bni1 (0.2×1000 molecules = 200 molecules) are on the membrane, and we estimate that ~50% of the actin are membrane-associated actin cables. The 50% is a rough estimate based on the observation that measurements in animal cells show that as much as 40% of actin in an animal cell is unpolymerized [17], and that phalloidin staining shows that polymerized actin is roughly evenly split between actin cables in the mating projection and actin patches in the cell body. For Spa2, Equation 6 yields:

$$\begin{aligned} \frac{Spa2_{\text{mss}}}{Spa2_{\text{t}}} &= \frac{S_{\text{on}}Actin_{\text{m}}}{S_{\text{off}} + S_{\text{on}}Actin_{\text{m}}}, \\ S_{\text{on}} = S_{\text{off}}\frac{Spa2_{\text{mss}}}{Spa2_{\text{t}}}\left[Actin_{\text{m}}(1 - \frac{Spa2_{\text{mss}}}{Spa2_{\text{t}}})\right]^{-1} &= \frac{0.35s^{-1} \times 0.9}{20 \text{ molecules } \times (1 - .9)}, \\ \Rightarrow S_{on} &= 0.158\,\text{molecules}^{-1}s^{-1}, \end{aligned} \tag{7}$$

where $Spa2_{\text{t}}$ is the total Spa2 population in the system. We made this fit with $Actin_m = 20$ because there is some evidence in the literature that this is roughly the correct value [13]. However, from the perspective of expected flux of Spa2 to the membrane we can linearly scale S_{on} for any expected $Actin_m$ to allow the first line of Equation 7 to hold. In fact, this is the procedure we performed to keep the expected membrane population of Spa2 constant as we varied the number of actin cables in the system to generate the phase planes in Figs. 6 and S16. Similarly, for Bni1 we have

$$\begin{aligned} \frac{Bni1_{\text{mss}}}{Bni1_{\text{t}}} &= \frac{B_{\text{on}}Cdc42_{\text{m}} + B_{\text{fb}}Spa2_{\text{mss}}}{S_{\text{off}} + B_{\text{on}}Cdc42_{\text{m}} + B_{\text{fb}}Spa2_{\text{mss}}}, \\ B_{\text{on}}Cdc42_{\text{m}} + B_{\text{fb}}Spa2_{\text{mss}} &= B_{\text{off}}\frac{Bni1_{\text{mss}}}{Bni1_{\text{t}}}\left[1 - \frac{Bni1_{\text{mss}}}{Bni1_{\text{t}}}\right]^{-1}. \end{aligned}$$

Finally, plugging in steady state values for Spa2 on the membrane and active Cdc42 yields:

$$\begin{aligned} B_{\text{on}}5400 \text{ molecules} + B_{\text{fb}}4500 \text{ molecules} &= \frac{0.25s^{-1} \times 0.20}{(1 - 0.20)} = 0.0625\,s^{-1}, \\ B_{\text{fb}} &= 1.39 \times 10^{-5}\,\text{molecules}^{-1}s^{-1} - 1.2B_{\text{on}}. \end{aligned} \tag{8}$$

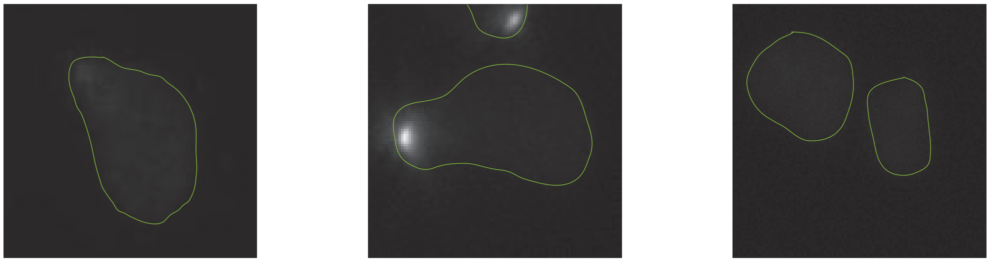

Figure S4: Quantifying the membrane-bound fraction of Bni1 and Spa2. In this Figure we show representative cells used to estimate the fraction of Bni1 and Spa2 on the cell membrane. The imaging parameters were the same for all images. The outlines of the cells are shown by the green line. The cells were segmented into the polarisome and the cytoplasm, and the fluorescence intensity was quantified for each. On the left is a typical cell containing Bni1-GFP which shows fluorescence both at the polarisome and in the cytoplasm. In the middle is a typical cell containing Spa2-GFP which exhibits bright polarisome fluorescence. On the right are two cells without any GFP, and we used these images to estimate the background autofluorescence. For Bni1-GFP and Spa2-GFP, we calculated the fraction of the total fluorescence intensity (after background subtraction) in the polarisome to be the membrane fraction.

3.6 $\mathbf{A_{on}}$

In [18] the authors use a heat sensitive mutant of Bni1 (which is known to be the formin responsible for nucleating actin cables during mating response). When these mutant cells are treated with heat, their Bni1 proteins lose function and no new actin cables are formed. Given that the cells were treated with heat when the polarisome was at steady state, the time evolution of the probability of each cable falling off is given by

$$A_{\mathrm{m}}(t) = A_{\mathrm{m}}(0)\left(1 - \exp^{OFF\times t}\right), \tag{9}$$

where $OFF = A_{\mathrm{off}}\frac{K_{\mathrm{m}}}{K_{\mathrm{m}}+Spa2_{\mathrm{local}}}$, $Spa2_{\mathrm{local}}$ is the population of Spa2 molecules available to stabilize actin cables at steady state, and $A_{\mathrm{m}}(0) = A_{\mathrm{mss}}$ is the number of actin cables on the membrane at steady state. As the actin cables fall off, the population of Spa2 on the membrane will decrease, however it is reasonable to assume that the steady state value is constant until that time. From fitting an exponential to the data in [18], we know that the time to half the steady state number of actin cables falling off the membrane is $\sim 45s$, thus, from Equation 9 we have

$$OFF = \frac{\log(2)}{45s}. \tag{10}$$

From SI Sections 3.5 and 3, we know that at steady state ~ 200 Bni1 molecules and $\sim 50\%$ of actin cables are on the membrane. We can write the following ODE for the total number of actin cables on the membrane:

$$\dot{A}_m = -OFFA_m + A_{\mathrm{on}}B_mA_c = -OFFA_m + A_{\mathrm{on}}B_m(A_{\mathrm{total}} - A_m). \tag{11}$$

At steady state, Equation 11 yields

$$A_{\text{on}} = \frac{OFF0.5}{Bm0.5} = \frac{\frac{\log(2)}{45s}}{200molecules} = 7.7016 \times 10^{-5} molecules^{-1}\, s^{-1}. \tag{12}$$

3.7 A_{off}

As mentioned above, we have the following relation:

$$A_{\text{off}} \frac{K_{\text{m}}}{K_{\text{m}} + Spa2_{\text{local}}} = \frac{\log(2)}{45s}, \tag{13}$$

where $Spa2_{\text{local}}$ is some Spa2 population close enough to a given actin cable to stabilize it. We know, in arbitrary units or normalized to a probability density function (PDF), the spatial location of Spa2 and Bni1 from experiments (for an example see Fig. S5).

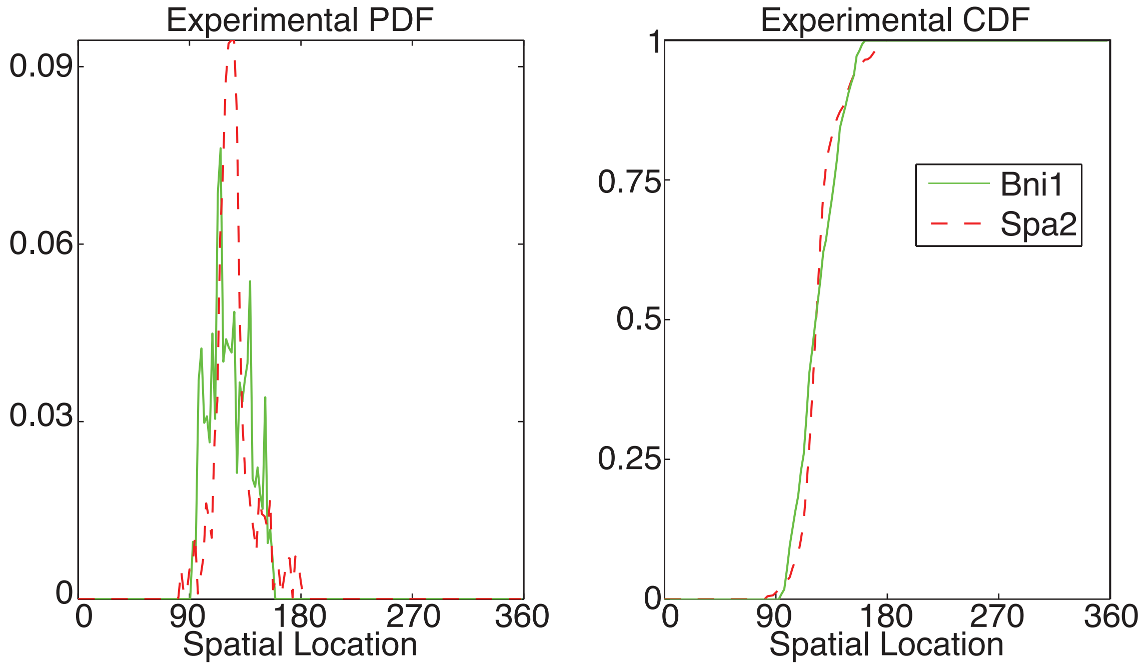

Figure S5: Example of converting spatial fluorescence intensity data to a PDF and then a CDF. **Green:** Bni1. **Red:** Spa2.

We know that at steady state, ~ 4500 Spa2 molecules are on the membrane. We also know that Bni1 nucleates actin cables, so the Bni1 distribution can be seen as an indicator of the probability of actin localization. The following procedure was used to simulate the experiment in [18]:

1. Distribute the Spa2 membrane population according to the experimental cumulative distribution function (CDF) for Spa2.
2. Distribute the actin membrane population according to the experimental CDF for Bni1.
3. Use these populations as the initial condition.
4. Run a stochastic simulation in which the only events are Spa2 leaving the membrane, Spa2 delivery to the membrane, lateral diffusion of Spa2 on the membrane and actin cable depolymerization (S_{off}, S_{on} and D take values as defined above, and A_{off} and K_{m} take values varied as described below).

5. The simulation is completed when half the actin cables have left the membrane and the final time is recorded.

This procedure was repeated for a given experimental data set, with the mean and standard error for the final time being updated after each run. The mean value was accepted once the relative size of the 95% confidence interval for the mean final time was $\leq 10\%$. For each data set this procedure is repeated for a range of K_{m} and A_{off} values. This is repeated for data sets from 10 different cells, and the results were then averaged (see Fig. S6).

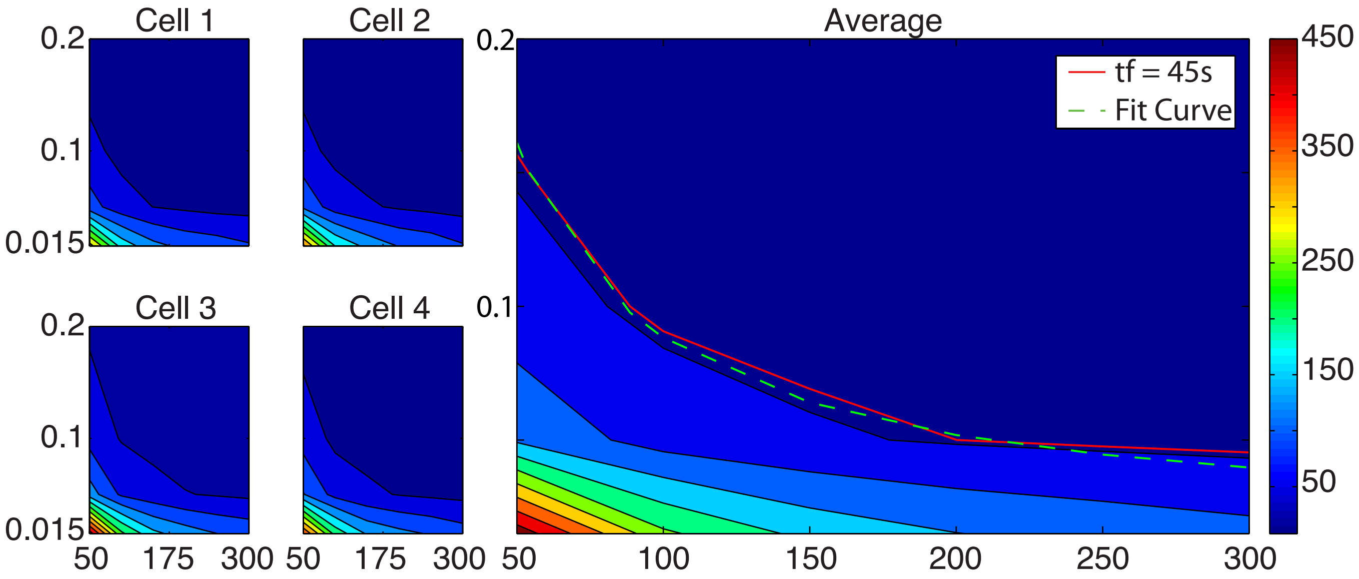

Figure S6: In all plots: **Vertical axis:** A_{off}. **Horizontal axis:** K_{m}. **Color bar:** seconds to removal of half the initial actin membrane population. Cells 1, 2, 3, 4 represent simulation results from 4 of the 10 data sets used. Average represents the average over the ten cells used. The red line on this plot represents the data points where the final time = 45s, and the green dashed line represents the fit to this curve.

Manipulating Equation 13 yields the following relationship between A_{off} and K_{m} for "time to half the actin cables off the membrane" $= 45$s:

$$\frac{45 \times A_{\mathrm{off}}}{\log(2)} - 1 = \frac{Spa2_{\mathrm{local}}}{K_{\mathrm{m}}}. \tag{14}$$

The only optimization variable is $Spa2_{\mathrm{local}}$, therefore we use linear regression to obtain the best linear unbiased estimate. The result is $Spa2_{\mathrm{local}} = 473$ (this is represented as the green dashed line in Fig. S6). Given that our model hypothesis is that Spa2 plays a role in stabilizing actin cables, this can be interpreted as stating that there are ~ 473 Spa2 molecules in the "neighborhood" of each actin cable to stabilize it.

3.8 Calculation of D

Reference [19] provides the following relation:

$$I(t) = I_{final}(1 - (w^2(w^2 + 4\pi Dt)^{-1})^{1/2}),$$

where w is the diameter of the area bleached, I_{final} is the max intensity and $I(t)$ is the intensity at time t. Taking $t = t_{1/2}$ (time to half recovery) yields:

$$D = \frac{3w^2}{4\pi t_{1/2}}. \tag{15}$$

4 Simulation of the Polarisome model

4.1 Stochastic Simulation Algorithm

Consider a system involving N molecular species $\{S_1, ..., S_N\}$, represented by the state vector $X(t) = [X_1(t), ..., X_N(t)]^T$, where $X_i(t)$ is the number of molecules of species S_i at time t. There are M reaction channels, labeled $\{R_1, ..., R_M\}$, in the system. Assume the system is well-mixed and in thermal equilibrium. The dynamics of reaction channel R_j are characterized by the *propensity function* a_j and by the *state change vector* $\nu_j = [\nu_{1j}, ..., \nu_{Nj}]^T$: $a_j(x)dt$ is defined as the probability that, given $X(t) = x$, one R_j reaction will occur in the next infinitesimal time interval $[t, t + dt]$, and ν_{ij} gives the change in X_i induced by one R_j reaction.

The system is a Markov process whose dynamics are described by the Chemical Master Equation (CME) [20]

$$\begin{aligned} \frac{\partial P\left(x, t|x_0, t_0\right)}{\partial t} &= \mathcal{M}\, P\left(x, t|x_0, t_0\right) \\ &= \sum_{j=1}^{M} \left[a_j(x - \nu_j)P(x - \nu_j, t|x_0, t_0) - a_j(x)P(x, t|x_0, t_0)\right], \end{aligned} \tag{16}$$

where the function $P(x, t|x_0, t_0)$ denotes the probability that $X(t)$ will be x, given that $X(t_0) = x_0$, and $\mathcal{M}$ denotes the generating matrix for the Markov chain that describes the chemical reactions. For all but the simplest systems, the chemical master equation is made up of an extremely large or infinite number (dimensions) of coupled ordinary differential equations (ODEs). Rather than evolve the CME directly, it is common practice to compute an ensemble of stochastic realizations whose probability density function converges to the solution of the CME. In chemical kinetics, the SSA [21] is used for this purpose.

At each step, the SSA generates two random numbers, r_1 and r_2 in $U(0, 1)$ (the set of uniformly distributed random numbers in the interval (0,1)). The time for the next reaction to occur is given by $t + \tau$, where τ is given by

$$\tau = \frac{1}{a_0} \ln\left(\frac{1}{r_1}\right) \tag{17}$$

where $a_0(x) = \sum_{j=1}^{M} a_j(x)$. The index μ of the occurring reaction is given by the smallest integer satisfying

$$\sum_{j=1}^{\mu} a_j > r_2 a_0. \tag{18}$$

The system states are updated by $X(t + \tau) = X(t) + \nu_\mu$. The simulation then proceeds to the time of the next reaction.

4.2 Inhomogeneous Stochastic Simulation Algorithm (ISSA)

Assume that the domain Ω in space is partitioned into voxels V_k, $k = 1, ..., K$. For simplicity of presentation, we will assume for the moment that the domain is in one dimension. Each molecular species in the domain is represented by the state vector $X_i(t) = [X_{i,1}(t), ..., X_{i,K}(t)]$, where $X_{i,k}(t)$ is the number of molecules of species S_i in voxel V_k at time t. Molecules in the domain are able to react with molecules within their voxel, as described above, and diffuse between neighboring voxels. The dynamics of diffusion of species S_i from voxel V_k to V_j is characterized by the *diffusion propensity function* $d_{i,k,j}$ and the *state change vector* $\mu_{k,j}$. $\mu_{k,j}$ is a vector of length K with -1 in the kth position, 1 in the jth position and 0 everywhere else: $d_{i,k,j}(x)dt$ gives the probability that, given $X_{i,k}(t) = x$, one molecule of S_i will diffuse from voxel V_k to V_j in the next infinitesimal time interval $[t, t + dt]$. Note that if $k = j \pm 1$ then $d_{i,j,k}(x) = D/l^2$, where D is the diffusion rate and l is the characteristic length of the voxel, and otherwise it is zero. The Diffusion Master Equation (DME) can then be written in a form similar to the CME:

$$\begin{aligned}\frac{\partial P\left(x,t|x_0,t_0\right)}{\partial t} &= \mathcal{D}\, P\left(x,t|x_0,t_0\right) \\ &= \sum_{i=1}^{N}\sum_{k=1}^{K}\sum_{j=1}^{K}\left[d_{i,j,k}(x_i-\mu_{k,j})P(x_1,...,x_i-\mu_{k,j},...,x_N,t|x_0,t_0) - d_{i,j,k}(x_i)P(x,t|x_0,t_0)\right], \quad (19)\end{aligned}$$

where $\mathcal{D}$ denotes the generating matrix for the Markov chain that describes the diffusion of molecules in the system.

The usual method of solution of the DME is to simulate each diffusive jump event explicitly, giving an exact solution. This is the method used by the ISSA and the next subvolume method (NSM) [22] algorithms.

Combining (16) and (19) yields the RDME

$$\frac{\partial P\left(x,t|x_0,t_0\right)}{\partial t} = \mathcal{M}P\left(x,t|x_0,t_0\right) + \mathcal{D}P\left(x,t|x_0,t_0\right). \quad (20)$$

The RDME is a linear constant-coefficient ODE, however it has many more possible states than the corresponding CME and thus is more difficult to solve. Rather than solve the RDME directly, it is common practice to compute an ensemble of stochastic realizations whose histogram converges to the probability distribution of the RDME. Actin cables do not diffuse on the membrane in our model, so their dynamics can be thought of as being described by the RDME with the diffusion coefficient set to zero (thus, there is localization without movement).

4.3 Spatial Deterministic Simulation Methods

The deterministic model is composed of two inhomogeneous parabolic partial differential equations and one ordinary differential equation:

$$\begin{aligned}\frac{\partial\left[Bni1_{\mathrm{m}}\right]}{\partial t} &= D\nabla^2 Bni1_{\mathrm{m}} + \left[Bni1_{\mathrm{c}}\right]\left(B_{\mathrm{on}}\left[Cdc42_{\mathrm{m}}\right] + B_{\mathrm{fb}}\left[Spa2_{\mathrm{m}}\right]\right) \\ &\quad - B_{\mathrm{off}}\left[Bni1_{\mathrm{m}}\right] \\ \frac{\partial\left[Spa2_{\mathrm{m}}\right]}{\partial t} &= D\nabla^2 Spa2_{\mathrm{m}} + S_{\mathrm{on}}\left[Actin_{\mathrm{m}}\right]\left[Spa2_{\mathrm{c}}\right] - S_{\mathrm{off}}\left[Spa2_{\mathrm{m}}\right] \\ \frac{\partial\left[Actin_{\mathrm{m}}\right]}{\partial t} &= A_{\mathrm{on}}\left[Bni1_{\mathrm{m}}\right]\left[Actin_{\mathrm{c}}\right] - A_{\mathrm{off}}\frac{K_{\mathrm{m}}}{K_{\mathrm{m}}+[X]}\left[Actin_{\mathrm{m}}\right]\end{aligned}$$

where X is $Spa2_{\mathrm{m}}$ for the S-model and $Bni1_{\mathrm{m}}$ for the B-model. We solved this system of equations numerically on the same spatial grid as the spatial stochastic simulation, with a timestep of 5e-4s utilizing the Crank-Nicolson method, an established stable solver for this class of problems [23].

5 Experimental Images and Modeling of Polarisome Dynamics

5.1 Myo2-Spa2 colocalization during the pheromone response

Previous work has suggested that Spa2 binds the actin motor protein myosin 2 (Myo2) [11]. In our model, we postulated that Spa2 travels along actin cables via Myo2 to be delivered to the polarisome. The addition of LatA results in the depletion of Spa2 from the membrane. To provide further evidence we examined their localization in a Myo2-GFP/Spa2-mCherry strain during the mating response. We observed strong colocalization of the two proteins at the polarisome after 2 hours of pheromone treatment (Fig. S7), and both proteins exhibit the fuzzy appearance found in proteins transported on actin fibers [24]. These data are consistent with the model that Spa2 binds Myo2 during projection formation and that both traverse actin cables to the polarisome.

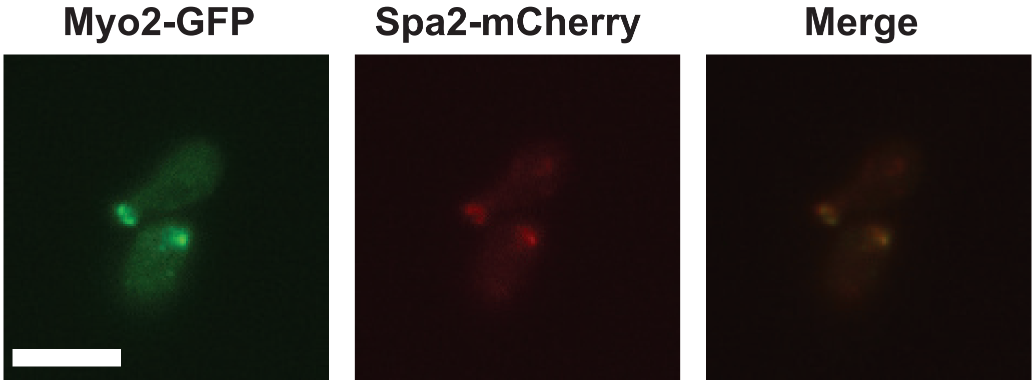

Figure S7: Colocalization of Myo2-GFP and Spa2-mCherry in the polarisome of pheromone-treated cells. Both proteins exhibit the fuzzy appearance at the periphery of the polarisome typical of proteins transported along the actin cables to the polarisome. Scale bar = 5 μm.

5.2 Reporters of active Cdc42

We used the fluorescent fusion protein Ste20-GFP as a reporter of active Cdc42 (Cdc42a). Ste20 is a PAK kinase that binds active Cdc42. It also contains a PIP2 binding domain that is essential for its localization. Importantly, both domains are necessary for proper localization: when either domain is deleted, Ste20 is not properly polarized [25]. Thus, binding of active Cdc42 is essential for membrane localization, and Ste20 is a good indicator of Cdc42a levels.

In the literature, the N-terminal CRIB domain of Gic2, Gic2-208, has also been used as a Cdc42a reporter [14], and we compared the two reporters. First, we investigated the colocalization of the two markers by constructing a strain containing Gic2-208-GFP/Ste20-mCherry, and then treated

cells with 1 μM alpha-factor for 2 hours. The two reporters polarized to the same region showing overlapping staining (Fig. S8A).

Second, we examined the spatial amplification in a dual-label Gic2-208-GFP/Spa2-mCherry strain (Fig. S8B). We found similar results to those from the Ste20-GFP/Spa2-mCherry strain. Thus, the degree of amplification of the polarisome is similar whether Cdc42a is measured by Ste20-GFP or by Gic2-208-GFP.

5.3 Polarisome Searching and Tracking Phenotypes

Fig. S9 shows the searching behavior of the polarisome. This behavior occurs on a much faster time scale (∼minutes) than the growth of the mating projection (∼hours). We hypothesize that this process integrates over time a noisy extracellular signal to increase the probability of finding a mating partner. Fig. S10 shows the repolarization behavior we refer to as tracking, using snapshot images and time series visualizations of Spa2-mCherry and Bni1-GFP during the repolarization process. Fig. S11 shows time series visualizations of the Spa2-mCherry and Ste20-GFP (Cdc42a reporter) of the tracking behavior. In addition, we note that the stochastic simulation dynamics in (Fig. 5D and 5F) are a better qualitative match to our experiments (Fig. 5C and 5E) than the deterministic simulations (Fig. S12A and 12B).

5.4 Tracking by the polarisome under gradient conditions

We examined the Ste20-GFP/Spa2-mCherry strain for polarisome tracking under both α-factor gradient (microfluidics) and isotropic (uniform) conditions. We observed the same basic behavior in both: First Ste20 (reporter of Cdc42a) shifted to the new second site, and then Spa2 tracked the directional change in Ste20. Fig S10 shows the results from a 10 - 100 nM gradient switch experiment in which the gradient was flipped 180°. We conclude that the dynamics of the polarisome tracking Cdc42a are similar under gradient or uniform conditions.

5.5 Tracking overlap time

We imaged cells with Bni1-GFP/Spa2-mCherry to observe the spatio-temporal dynamics of these two polarisome components during tracking. There is a $\sim$ 10 minute transition period during which the nascent second polarisome has begun to form while the initial polarisome still persists. We constructed a boxplot of these overlap times from *in vivo* experiments and *in silico* simulations (see Fig. S13).

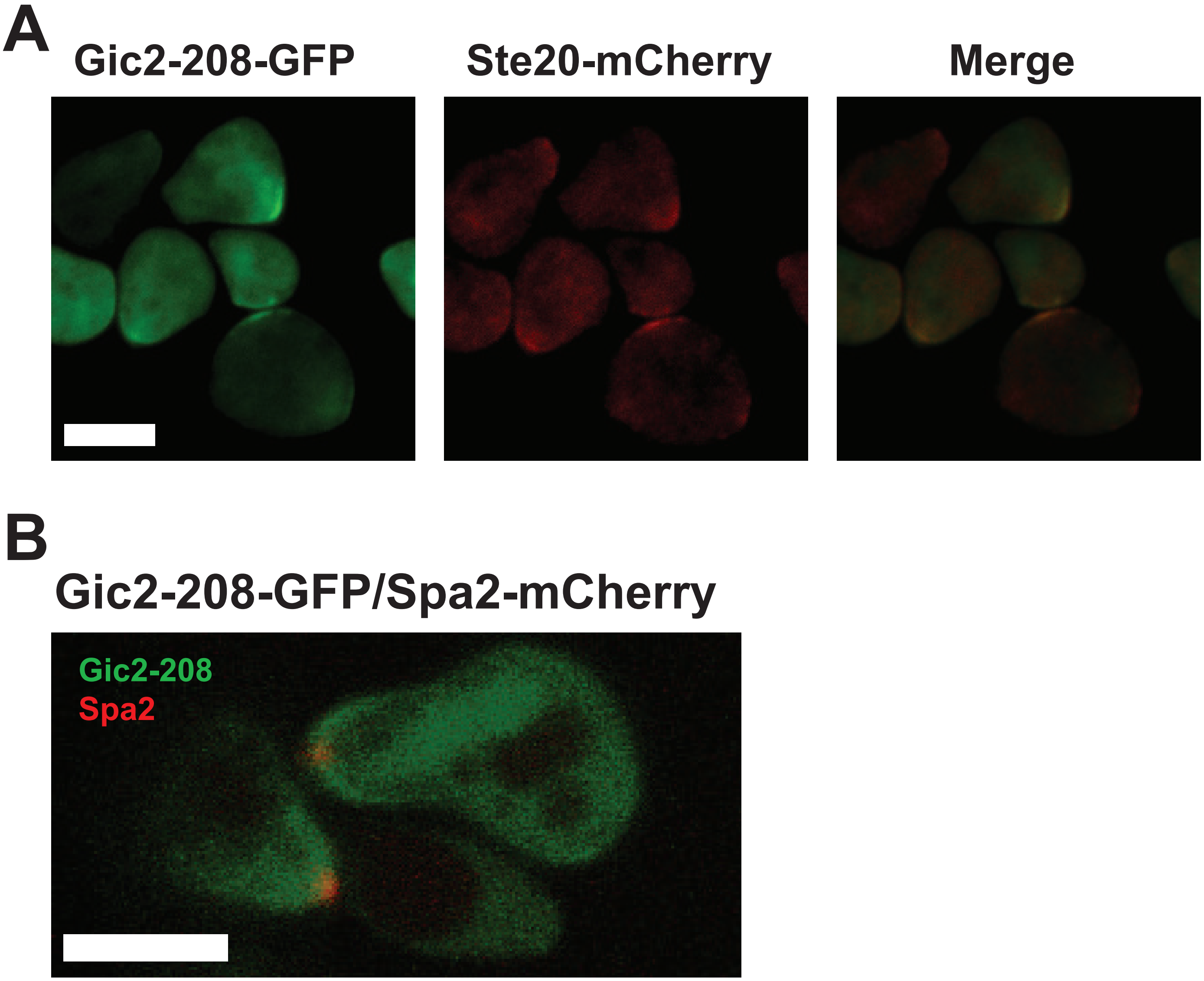

Figure S8: Equivalence of Ste20 and Gic2-208 as reporters for active Cdc42. **(A)** Cells containing both Gic-208-GFP and Ste20-mCherry were treated with α-factor for two hours and imaged. The two reporters colocalized to the polarized region in the mating projection. **(B)** Spatial amplification of Spa2-mCherry in Gic2-208-GFP strain. An equivalent level of Spa2 amplification was observed in the Gic2-208-GFP strain as in the Ste20-GFP strain. Scale bar = 5 μm.

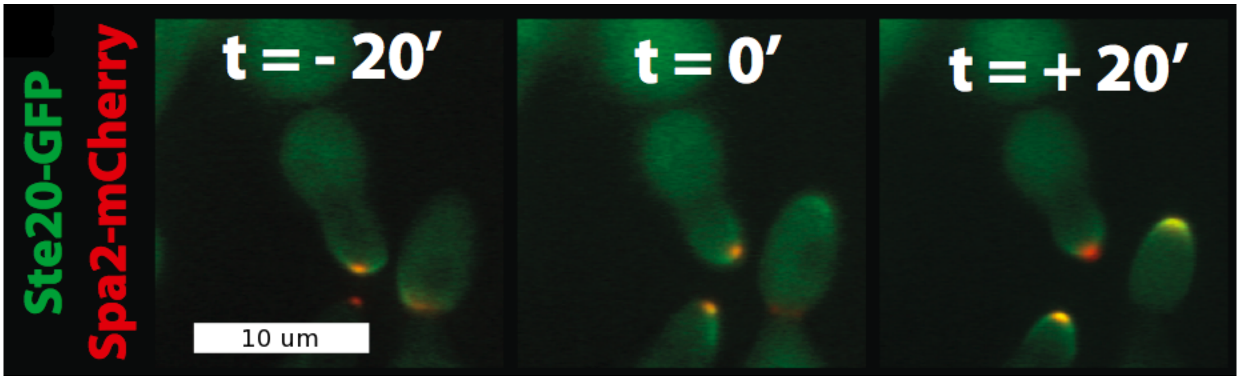

Figure S9: Experimental evidence of searching. Over the course of a 40 minute observation (in a solution of 10 nM alpha factor), we see the polarisome (small red/orange region on the lower edge of the center cell) shift to the left (left frame), to the right (center frame) and to the center (right frame) of the polarized region. This dynamic localization of the polarisome, which we refer to as the searching phenotype, may help the cell find a mating partner in a noisy and changing environment.

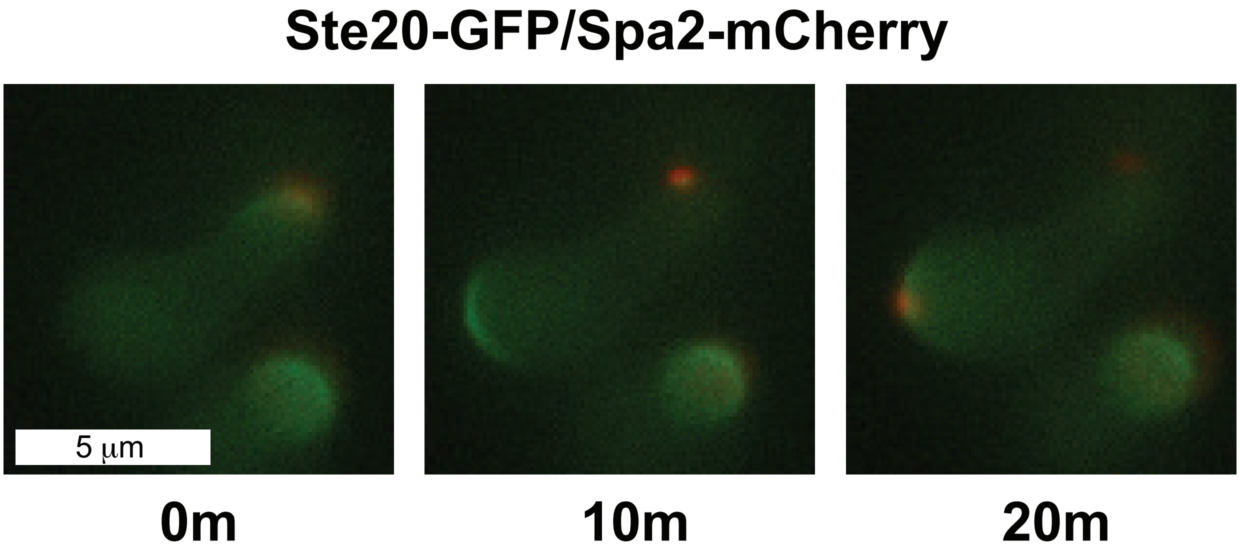

Figure S10: Ste20-GFP/Spa2-mCherry strain was subjected to a 10 nM - 100 nM gradient switch experiment. Cells were imaged at 10 min intervals after the 180° change in the gradient direction. As is the case under isotropic pheromone treatment, Ste20 moves to the new site first, and then Spa2 tracks the directional change in Cdc42a (Ste20). Scale bar = 5 μm.

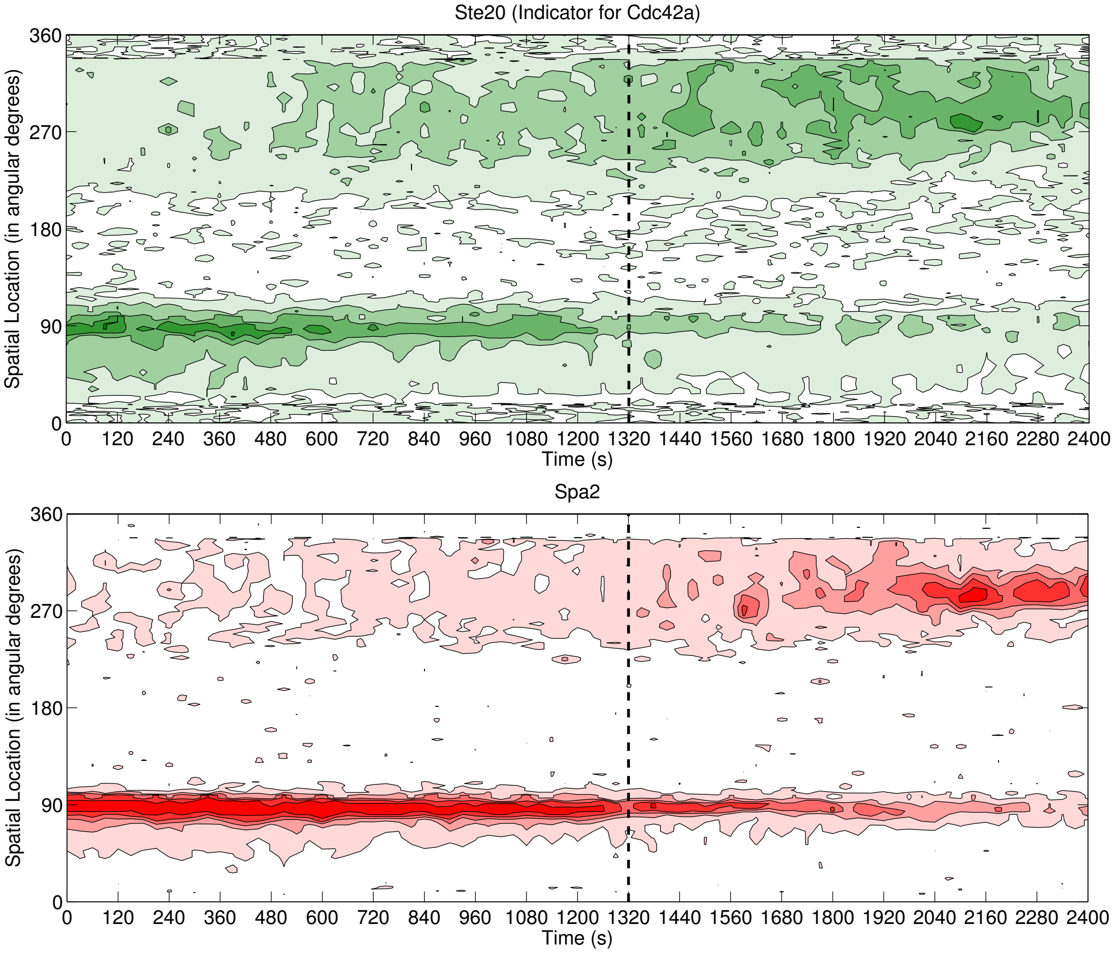

Figure S11: Time series data for Ste20 and Spa2 during repolarization. **Top:** Time series visualization of Ste20 (marker for Cdc42a) in green from the experimental results shown in Figure 3A in the main text. **Bottom:** Time series visualization of Spa2 (in red) from the experimental results shown in Figure 3A in the main text (black dashed line indicates time of Cdc42a switch).

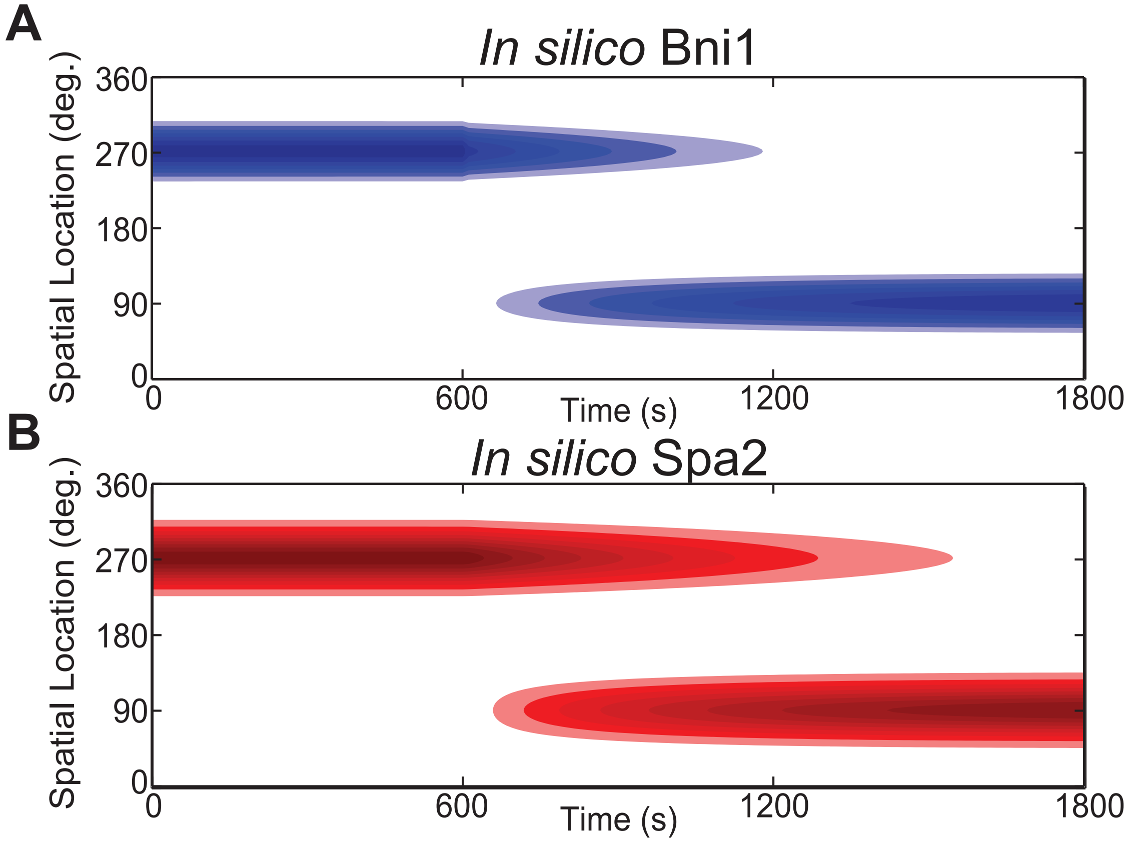

Figure S12: Deterministic Simulation: Spatial dynamics of Bni1 **(A)** and Spa2 **(B)** during polarisome tracking of Cdc42a. Compare with Figures 5C, D, E and F.

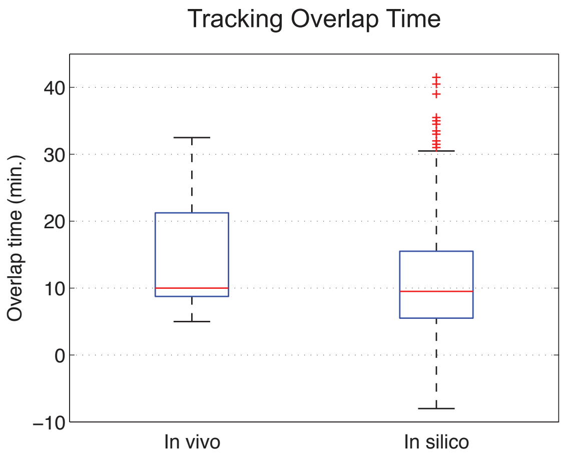

Figure S13: Boxplot of Spa2 overlap time: Overlap time is the length of time during tracking when the a old polarisome has not disappeared but the nascent polarisome has begun. These boxplots represent data from live cell imaging and simulations. A representative time series of this data can be seen in Figure 5.

6 Measuring the Polarisome Width

Fig. S14A shows a box plot representation of polarisome width measurements for Ste20 (indicating active Cdc42) and Spa2 *in vivo*. Ste20-GFP/Spa2-mCherry cells were treated with 20 nM α-factor for 2 h and then fixed in formaldehyde. Our measurements indicate a mean FWHM for Ste20 of 48° and for Spa2 of 18°. Fig. S14B shows a corresponding boxplot of the FWHM of Spa2 from our stochastic simulations.

We note here that there is a difference between the mean width presented in Fig. S14 and that in Fig. 4D (black diamonds). The difference comes from measuring the width at individual time points (which is the data in Figs. S14A and B) versus measuring the width of all the data points summed together (which is the wider distribution in Fig. 4D). Another way to think about this distinction is the difference in the order of operations: in the first case we found the width at each time point, then took the average of those widths. In the second we took the average over all time points then measured the width. If the polarisome were stationary (as in the deterministic case) these two widths would be the same, but if it is moving around the second will be broader. Videos S7 and S8 show *in vivo* and *in silico* evidence of this movement or 'searching' behavior of the polarisome. If you look at any one frame you will see a tight polarisome, with a width of $\sim$ 18 degrees. However, as you watch the video you will see the polarisome shift or 'search' along the Cdc42a profile, and if you take a time or ensemble average you get a wider distribution that looks a lot like the Cdc42a and the deterministic Spa2 profiles.

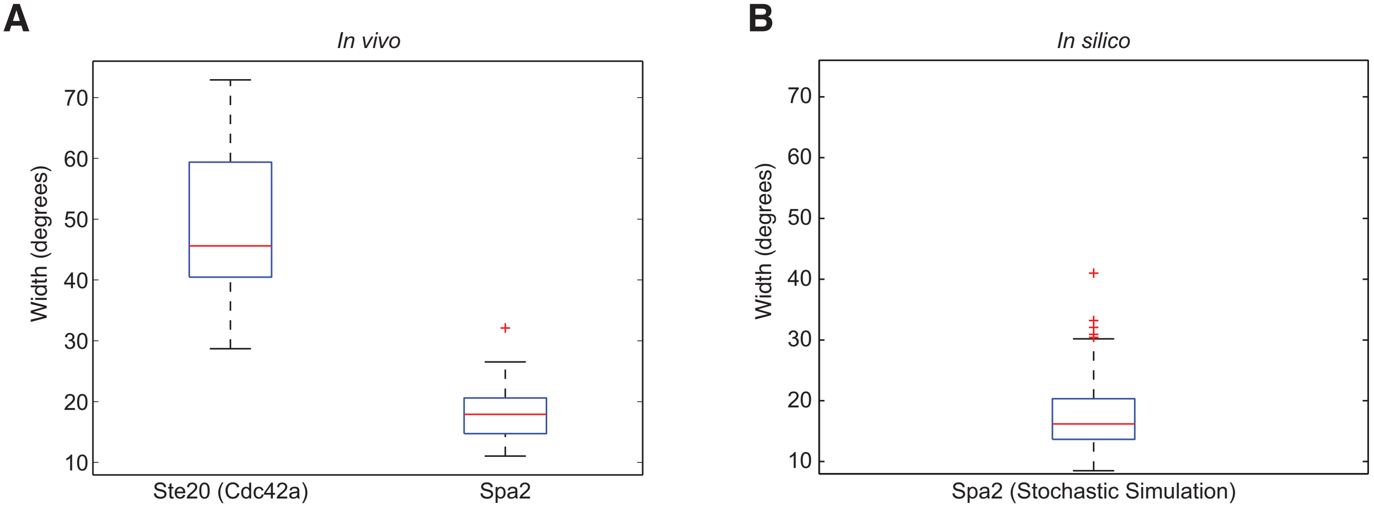

Figure S14: *in vivo* and *in silico* measurements of polarization width. **(A)** Box-plots of measurements of polarization width for **left:** Ste20 (indicator for Cdc42a, left) and **right:** Spa2 from 31 *in vivo* fixed yeast cells. **(B)** Box-plot of measurements of polarization width for Spa2 from 31 *in silico* cells with the nominal parameter set in Table S4.

7 Phenotype Space Plots

The relative strength of B_{on} (which represents sensing of the the input, Cdc42a) and B_{fb} (which represents positive feedback and self-sustaining polarisome growth) determines the degree to which the polarisome reacts to its environment in the model. B_{on} is a key parameter that modulates the interaction between the input profile (Cdc42a) and the rest of the model components. It is also independent of the actin-Spa2 positive feedback loop. As such, it provides spatial cues for polarisome formation. The opposing force to the cue provided by B_{on} is B_{fb}. B_{fb} represents the strength of the positive feedback, (which represents the independence of polarisome's shape from the input signal). The balance of these two parameters determine whether the simulation will be in a regime where the output width and strength is independent of but directionally biased by the input signal ($B_{\text{on}} < B_{\text{fb}}$), or dependent on the input signal ($B_{\text{on}} > B_{\text{fb}}$).

The other free parameter is K_{m}, which dictates the stability of actin cables. At lower K_{m}, the actin cables are more stable. This stability lends itself to strong polarisome formation, but it also reduces the ability of the polarisome to respond to changes in the external gradient. To understand the impact of these parameters on polarization, we used two metrics to characterize the simulation results: the polarization width of Spa2 and the probability of tracking an input signal that changes direction by 180°. These metrics are described in the main text.

Note that the K_{m} values for the stochastic model and the K_{m} values for the deterministic model have the same units and are directly comparable. For the deterministic simulations, we multiplied all concentrations by the voxel volume to obtain numbers of molecules per voxel, which is the same units as the stochastic simulations.

The polarization phenotype space plots in Fig. S15 illustrate the differences in behavior between the deterministic and stochastic simulations of the polarisome model. For every parameter set the stochastic simulation produced narrower polarisomes than the deterministic equivalent. As expected, both descriptions have a higher probability of tracking for lower $B_{\text{fb}}/B_{\text{on}}$ (more directional information from the input) and larger K_m (actin cables are less stable). The stochastic case (Fig. S15A) exhibited a graded tracking response which allows for perturbations that will lead to a change in the probability but not to complete tracking failure. In the deterministic case (Fig. S15B), tracking was seen to be a switch-like behavior, in which small perturbations in parameter values near the transition can lead to a complete failure to track. Thus the stochastic formulation of the model exhibits more robust tracking.

The differences in the region of successful tracking became more exaggerated when we examined polarisome width (Fig. S15C and D). In both models, the width decreased with higher $B_{\text{fb}}/B_{\text{on}}$ (moving toward input independent width) and smaller K_{m} (actin cables are more stable). Finally, we observe that for wild-type polarisome width (FWHM $\sim 18°$), the stochastic simulations yield a high probability of tracking whereas the deterministic simulations exhibit no tracking for many parameter values along the contour line.

Similar results are observed in the B-model (Fig. S17).

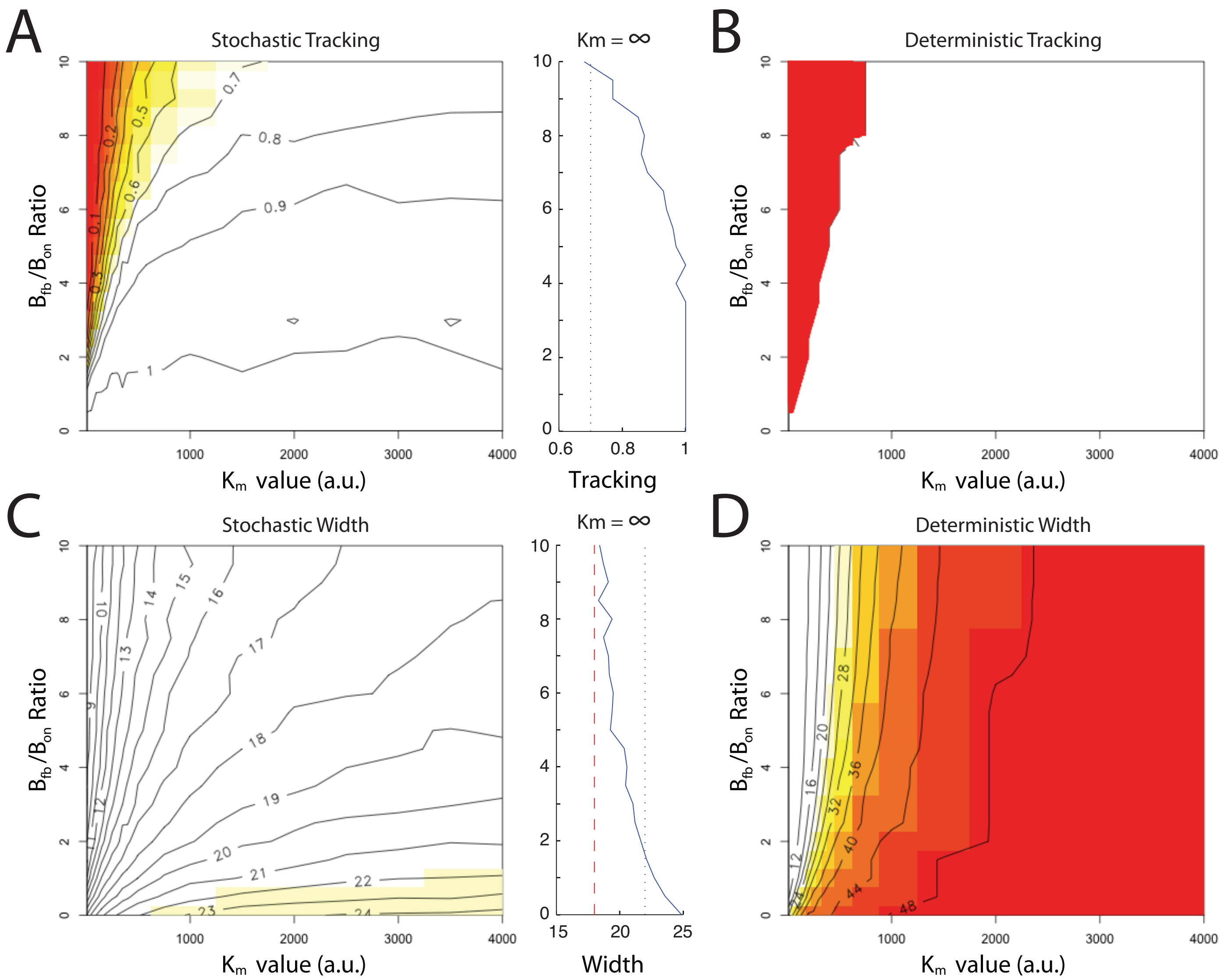

Figure S15: Polarization phenotype space plots of Spa2 in the S-model showing polarisome tracking and width for the two model parameters, $B_{\text{fb}}/B_{\text{on}}$ and K_{m}. Red is worse performance (lower probability of tracking, wider FWHM), white is better performance (higher probability of tracking, narrower FWHM). The remaining free parameters $B_{\text{fb}}/B_{\text{on}}$ (ratio of feedback to spontaneous association of Bni1) and K_{m} (stability of actin cables) represent the two trade-offs in polarization behavior: input sensing vs. feedback and stability. **(A)** Stochastic Tracking. The right side of this plot shows results for $K_m = \infty$ (blue line) and the cut-off for sufficient tracking (vertical dashed black line, 70% probability). **(B)** Deterministic Tracking. **(C)** Stochastic Width. The right side of this plot shows results for $K_m = \infty$ (blue line) and the cut-off for sufficiently narrow polarisome width (vertical dashed black line, $22°$ width). Note that the average simulation width for $K_m = \infty$ was greater than the average experimental width (vertical dashed red line, $18°$ width). In contrast, a width of $18°$ was easily achieved when $K_m < \infty$. **(D)** Deterministic Width. The tracking panels (A,B) show the probability that the polarisome is within 90° of the input signal after an initial polarization and then changing of the signal direction by 180°. The width panels (C,D) show the full width at half max of a circular Gaussian function fit to the data. For the stochastic simulation, each point represents the mean of 500 trajectories. The simulations were run for $2040s$, with the location of max input moving 180 degrees at $t = 1020s$.

8 Simulation results for B-Model

As discussed previously, we explored an alternate version of the polarisome model with Bni1 stabilizing actin cables (B-model) instead of Spa2 (S-model). Since there is less Bni1 than Spa2 in the system we had to adjust the K_{m} values used for the S-model to explore the equivalent range of the parameter space. To do this, we used the formula $K_{\mathrm{m}}^{\text{B-model}} = (.2Bni1_t)/(.9Spa2_t)K_{\mathrm{m}}^{\text{S-model}}$. Thus our nominal parameter set for the B-model is $K_{\mathrm{m}} = 155$, and $B_{\mathrm{fb}}/B_{\mathrm{on}}$ ratio=7.5 (same as S-model). This section shows results for the B-model that are equivalent to the S-model results shown previously. Fig. S16 shows five stochastic polarization phenotype space plots for increasing number of actin cables and one deterministic, similar to Fig. 6. Fig. S17 shows the detailed contour phenotype space plots, similar to Fig. S15. Fig. S18 shows the simulation results comparing wild-type vs. spa2Δ mutant phenotypes for both stochastic and deterministic models, similar to Fig. 7.

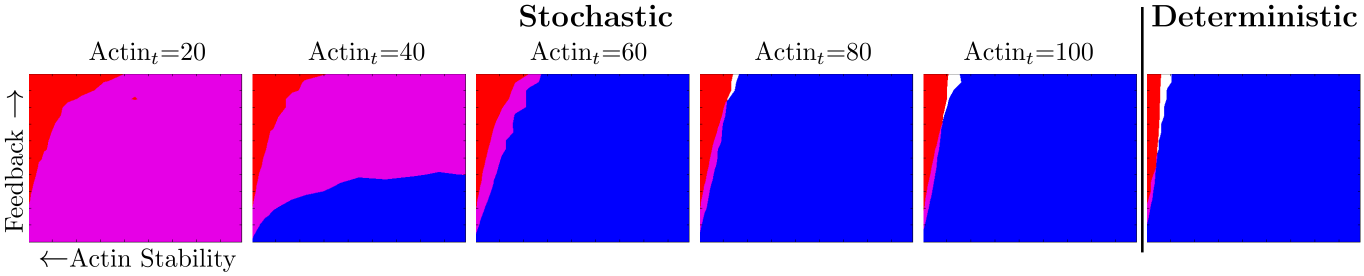

Figure S16: Six polarization phenotype space plots of $B_{\mathrm{fb}}/B_{\mathrm{on}}$ ratio vs. K_{m} using the B-model. The first five panels show results from the stochastic model with varying Actin$_{\mathrm{t}}$ values of 20, 40, 60, 80, 100 (left to right); the final panel shows results from the deterministic model. K_{m} values (x-axis) range from 0 to 175, $B_{\mathrm{fb}}/B_{\mathrm{on}}$ ratio (y-axis) ranges from 0 to 10. Blue indicates accurate tracking (>70% probability), red indicates narrow width (<22° FWHM), and purple indicates that both criteria are met.

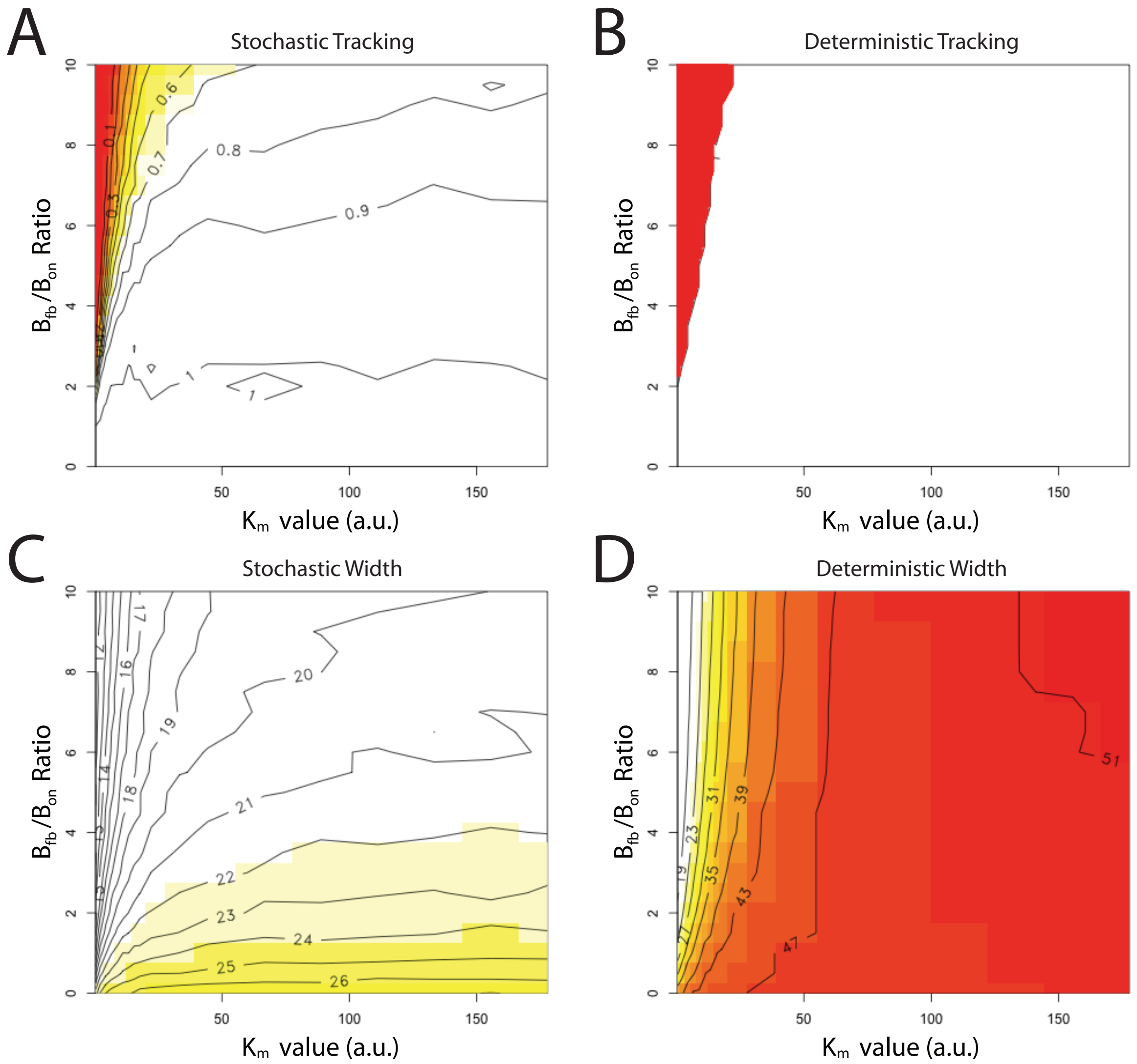

Figure S17: Polarization phenotype space plots of Spa2 in the B-model showing polarisome tracking and width for the two model parameters, $B_{\text{fb}}/B_{\text{on}}$ and K_{m}. Red is worse performance (lower probability of tracking, wider FWHM), white is better performance (higher probability of tracking, narrower FWHM). The remaining free parameters $B_{\text{fb}}/B_{\text{on}}$ (ratio of feedback to spontaneous association of Bni1) and K_{m} (stability of actin cables) represent the two trade-offs in polarization behavior: input sensing vs. feedback and stability. **(A)** Stochastic Tracking. **(B)** Deterministic Tracking. **(C)** Stochastic Width. **(D)** Deterministic Width. The tracking panels (A,B) show the probability that the polarisome is within 90° of the input signal after an initial polarization and then changing of the signal direction by 180°. The width panels (C,D) show the full width at half max of a circular Gaussian function fit to the data. For the stochastic simulation, each point represents the mean of 500 trajectories. The simulations were run for $2040s$, with the location of max input moving 180 degrees at $t = 1020s$.

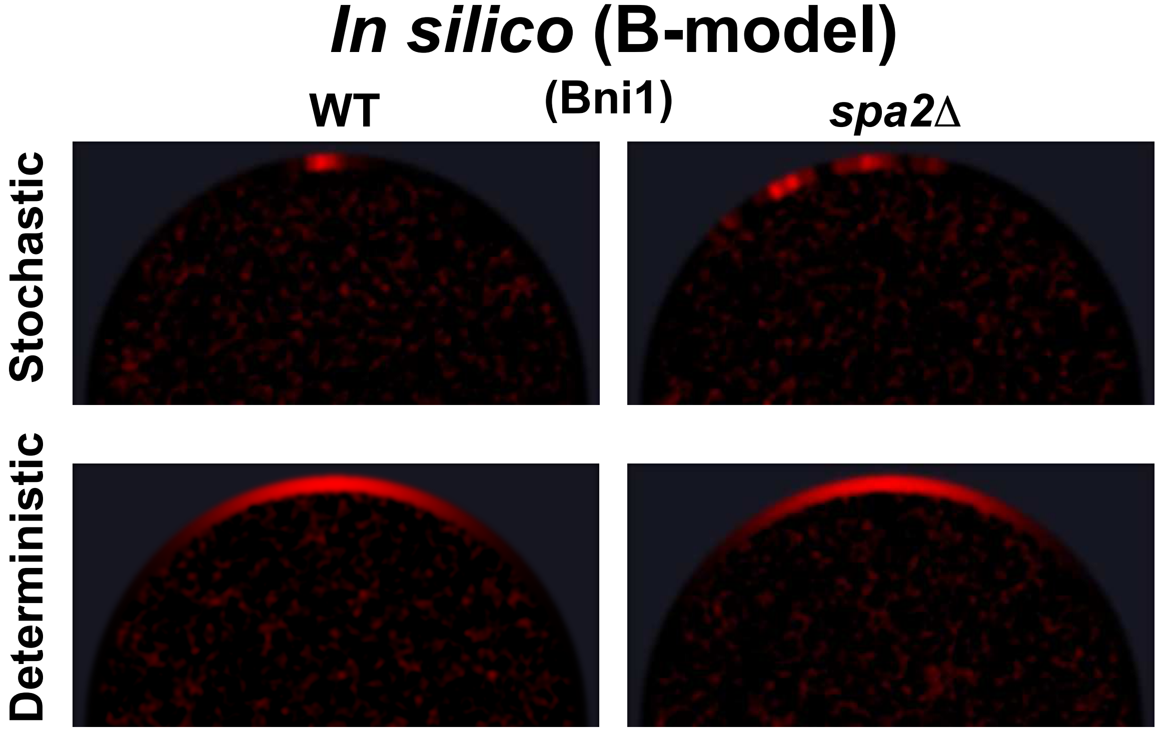

Figure S18: The multi-polarisome phenotype in *spa2*Δ cells. **Columns:** WT phenotype (**left**), *spa2*Δ phenotype (**right**). *In silico* snapshots of yeast polarisome simulations using the B-Model for both stochastic (top row) and deterministic (bottom row) models. Like the results from the S-model, only the stochastic *in silico* B-model is able to match the *in vivo* multi-polarisome phenotype (See Fig. 7 in the main text). The simulation parameter values were K_{m}=155, $B_{\mathrm{fb}}/B_{\mathrm{on}}$ ratio = 7.5.

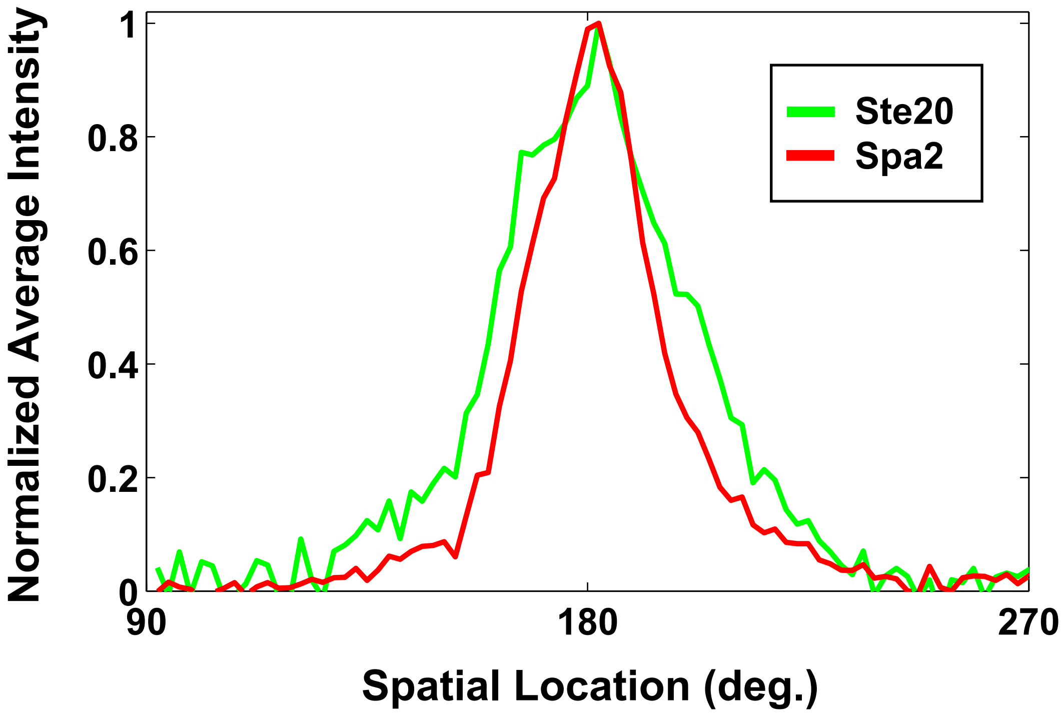

Figure S19: Ensemble average of experimental polarization data. Time-lapse images of Ste20-GFP/Spa2-mCherry cells were taken. The fluorescence intensities were averaged over frames and cells. The average width for Ste20 was 40.5°, and the average width of Spa2 was 32.6°. By taking the ensemble average, we found that the Spa2 data smeared out in different directions resulting in a broader peak as was observed in the simulations. However, it did not average to the Ste20 input profile. Polarisome movement was biased toward the center compared to the simulations.

References

[1] Sheu YJ, Barral Y, Snyder M (2000) Polarized growth controls cell shape and bipolar bud site selection in *Saccharomyces cerevisiae.* Mol Cell Biol 20: 5235-5247.

[2] Pruyne D, Bretscher A (2000) Polarization of cell growth in yeast: I. Establishment and maintenance of polarity states. J Cell Sci 113: 365-375.

[3] Köhli M, Galati V, Boudier K, Roberson R, Philippsen P (2008) Growth-speed-correlated localization of exocyst and polarisome components in growth zones of *Ashbya gossypii* hyphal tips. J Cell Sci 121: 3878-3889.

[4] Jones L, Sudbery P (2010) Spitzenkörper, exocyst, and polarisome components in *Candida albicans* hyphae show different patterns of localization and have distinct dynamic properties. Eukaryotic Cell 9: 1455-1465.

[5] Layton AT, Savage NS, Howell AS, Carroll SY, Drubin DG, et al. (2011) Modeling vesicle traffic reveals unexpected consequences for Cdc42p-mediated polarity establishment. Current Biology 21: 184 - 194.

[6] Buttery SM, Yoshida S, Pellman D (2007) Yeast formins Bni1 and Bnr1 utilize different modes of cortical interaction during the assembly of actin cables. Mol Biol Cell 18: 1826-1838.

[7] Evangelista M, Blundell K, Longtine MS, Chow CJ, Adames N, et al. (1997) Bni1p, a yeast formin linking Cdc42p and the actin cytoskeleton during polarized morphogenesis. Science 276: 118-122.

[8] Marco E, Wedlich-Soldner R, Li R, Altschuler S, Wu L (2007) Endocytosis optimizes the dynamic localization of membrane proteins that regulate cortical polarity. Cell 129: 411 - 422.

[9] Sanft K, Gillespie D, Petzold L (2011) Legitimacy of the stochastic michaelis??menten approximation. Systems Biology, IET 5: 58 -69.

[10] Goode BL, Eck MJ (2007) Mechanism and function of formins in the control of actin assembly. Annual Review of Biochemistry 76: 593-627.

[11] Shih JL, Reck-Peterson SL, Newitt R, Mooseker MS, Aebersold R, et al. (2005) Cell polarity protein Spa2p associates with proteins involved in actin function in *Saccharomyces cerevisiae.* Mol Biol Cell 16: 4595-4608.

[12] Yi TM, Kitano H, Simon MI (2003) A quantitative characterization of the yeast heterotrimeric G protein cycle. Proc Natl Acad Sci U S A 100: 10764-9.

[13] Yu J, Crevenna A, Bettenbuhl M, Freisinger T, Wedlich-Soldner R (2011) Cortical actin dynamics driven by formins and myosin V. J Cell Sci 124: 1533-1541.

[14] Ozbudak EM, Becskei A, van Oudenaarden A (2005) A system of counteracting feedback loops regulates Cdc42p activity during spontaneous cell polarization. Developmental Cell 9: 565 - 571.

[15] Wedlich-Soldner R, Altschuler S, Wu L, Li R (2003) Spontaneous cell polarization through actomyosin-based delivery of the Cdc42 GTPase. Science 299: 1231-1235.

[16] Elowitz MB, Surette MG, Wolf PE, Stock JB, Leibler S (1999) Protein mobility in the cytoplasm of *Escherichia coli.* Journal of Bacteriology 181: 197-203.

[17] Lodish H, Berk A, Kaiser C, Krieger M, Scott M, et al. (2000) Molecular Cell Biology. New York: W.H. Freeman, 4th edition.

[18] Evengelista M, Pruyne D, Amberg DC, Boone C, Bretscher A (2002) Formins direct Arp2/3-independent actin filament assembly to polarize cell growth in yeast. Nat Cell Biol 4: 32-41.

[19] Lippincott-Schwartz J, Presley J, Zaal K, Hirschberg K, Miller C, et al. (1998) Chapter 16: Monitoring the dynamics and mobility of membrane proteins tagged with green fluorescent protein. In: Sullivan KF, Kay SA, editors, Green Fluorescent Proteins, Academic Press, volume 58 of *Methods in Cell Biology.* pp. 261 - 281.

[20] Gillespie D (1992) A rigorous derivation of the chemical master equation. Physica A: Statistical Mechanics and its Applications 188: 404 - 425.

[21] Gillespie D (1977) Exact stochastic simulation of coupled chemical reactions. The Journal of Physical Chemistry 81: 2340–2361.

[22] Elf J, Ehrenberg M (2004) Spontaneous separation of bi-stable biochemical systems into spatial domains of opposite phases. Systems Biology, IEE Proceedings 1: 230-236.

[23] Crank J, Nicolson P (1996) A practical method for numerical evaluation of solutions of partial differential equations of the heat-conduction type. Advances in Computational Mathematics 6: 207-226.

[24] Fehrenbacher K, Huckaba T, Yang HC, Boldogh I, Pon L (2003) Actin comet tails, endosomes and endosymbionts. Journal of Experimental Biology 206: 1977-1984.

[25] Takahashi S, Pryciak P (2007) Identication of novel membrane-binding domains in multiple yeast Cdc42 effectors. Mol Biol Cell 18: 4945-4956.
